# Impact of the EU Blue Card programme on cultural participation and subjective well-being of migrants in Germany

**DOI:** 10.1371/journal.pone.0253952

**Published:** 2021-07-12

**Authors:** Eleftherios Giovanis, Sacit Hadi Akdede, Oznur Ozdamar

**Affiliations:** 1 Department of Public Finance, Nazilli Faculty of Economics and Administrative Sciences, Aydin Adnan Menderes University, İsabeyli/Nazilli/Aydın, Turkey; 2 Department of Economics, Faculty of Economics and Administrative Sciences, Izmir Bakircay University, Menemen, İzmir, Turkey; Universitat de Valencia, SPAIN

## Abstract

The first aim of this study is to investigate the role of the EU Blue Card programme implemented in 2012 in Germany. In particular, we aim to explore the impact on the participation in cultural activities of first-generation non-European Union (EU) and non-European Economic Area (EEA) migrants, such as attendance to cinema, concerts and theatre. The second aim is to examine the impact of cultural activities on subjective well-being (SWB), measured by life satisfaction. We compare the cultural participation and life satisfaction between the treatment group that is the non-EU/EEA first-generation immigrants and the control group that consists, not only of natives and second-generation immigrants but also composes of EU/EEA first-generation immigrants who are not eligible to the programme. We will apply a sharp and a fuzzy regression discontinuity design (RDD) within a seemingly unrelated regression equations (SURE) system using the Ordered Probit method. The empirical analysis relies on data from the German Socio-Economic Panel (GSOEP) survey over the period 2015–2018. The results show that the treated subjects experience an increase in cultural participation activities and an improvement in their SWB, as a result of the EU Blue Card programme, compared to the control group. Participation in classical music performance, opera or theatre influences more the SWB compared to other cultural activities. Policies that promote labour market integration and participation in cultural activities will enable immigrants to integrate into the social norms of the host societies and improve their SWB.

## Introduction

Labour and refugee migration have long been an integral and significant part of the history of Europe. Nonetheless, Europe’s recognition of migration as an important concern for society is relatively recent. For years, countries in Europe and across the globe have adopted different migration policies aimed at the economic and political integration and social inclusion of migrants. Despite the extensive evidence in the literature on the determinants of socio-economic and political integration and the effects of relevant migration policies on integration, little attention has been paid to research about the role of such policies in the cultural participation of migrants and the relationship between SWB and participation in cultural activities.

Cultural engagement, due to its potential individual and social impact on well-being, especially in recent years, is one of the most discussed human rights concerns [1]. The relation of cultural engagement and subjective well-being (SWB) has been studied in social intervention and public policies, because of the belief that this facility brings into social inclusion and reduces social isolation and exclusion, building stronger and cohesive communities, which in turn enhances overall human development and well-being [2, 3]. The relationship between well-being and cultural participation has been understudied according to some scholars [2], although other scholars argue that the findings are incomplete and inconclusive [3, 4].

According to the UNESCO definition [5], cultural participation can be defined as participation in any activity that increases the cultural and knowledge capacity and the capital of individuals that helps them develop their own identity and enables them to express themselves. Activities of this type can take various forms, both active, such as participating in artistic practising activities, a theatre production, or composing music, and passive participation, such as attending a cinema or a concert. It is important to explore the influence of migration policies on cultural participation and analyse the relationship between cultural activities and SWB, as the latter may act as a key driver of human capital to increase productivity and social inclusion, and boost overall development. The majority of the earlier studies have explored economic, demographic, political and institutional outcomes as measures of integration, including wages, employment, language, citizenship rights, religion, fertility choices and language [6–12]. While few studies have explored the role of different types of cultural and leisure activities as part of the integration process [13, 14], our study contributes to the earlier literature by exploring the impact of the migration policy of the EU Blue Card programme in 2012 on cultural participation using a sharp regression discontinuity design (RDD) and as a robustness check, we apply a fuzzy RDD. While we recognize that integration is very important for promoting social inclusion and the well-being of migrants in terms of economic and political initiatives, such as citizenship rights, employment and wages, we argue that research should also focus on the effect of policies on cultural participation, as it can demonstrate well-being, promote social inclusion and build a space for cross-cultural dialogue [15, 16].

The first aim of this study is to explore the impact of the EU Blue Card programme implemented in 2012 in Germany on participation in cultural activities of first-generation non-EU/EEA migrants. Cultural activities are operationalized by attendance at cinema and jazz or pop concerts; attendance at a classical music performance, theatre or opera; and practising artistic activities. The second aim is to explore the impact of those cultural activities on the subjective well-being expressed by life satisfaction.

The remainder of this paper is divided into five sections: In section 2 we describe the EU Blue Card programme. In section 3 we present the data and methods employed in the empirical work. In section 4 we present the empirical results and in section 5 we discuss the concluding remarks of the study’s findings.

## The EU Blue Card programme

Since August 1, 2012, the “EU Blue Card” has been in effect, and this programme is a key process to focus on a selective migration strategy, in which its implementation should facilitate access to the labour market for highly skilled non-EU/EEA nationals and, in particular, for those who have completed a university degree. This is achieved by an initially restricted entry without a “priority check”, which is to control whether German natives or EU nationals are eligible for the particular job position in the case of an offer of employment and a gross annual salary above 44,800€. In addition, if the employment offer is in a high demand profession, and more precisely, whether it refers to natural scientists, engineers, physicians and computer scientists, then the earning threshold is set up at 34,944€ [17].

The “EU Blue Card” deal, accompanied by language and cultural orientation courses, includes a work permit for two to four years in an EU country. As regards social legislation and employment law, the owner of such a card must be considered a resident, while the work permit is obtained in parallel by relatives. Applicants may be granted a settlement permit after three years, which is similar to a permanent residence permit, while, if the applicants can demonstrate German language skills at B1 level, they may be granted a settlement permit after two years, provided that the applicants do not lose their employment. This, in turn, implies that if the applicants lose their employment during that initial period, then they must leave the EU. For companies with a demand for a highly qualified workforce, such as technology-oriented or knowledge-based small-medium enterprises (SMEs), the provision is mainly important [17]. The recognition of foreign education certificates was simplified on 1^st^ April 2012 and, such as procedure, a process for reviewing and comparing international education certificates against German certificates was implemented. A formal assessment is undertaken as to whether training is equivalent to training in Germany and whether qualifications can be accepted and recognized [18].

However, we should notice that the minimum salary for both cases does not remain constant across the period we examine. In particular, the thresholds of 44,800€ and 34,944€ refer to 2012, the first year of the EU Blue Card programme implementation, while an annual increase in the minimum salaries is taking place as we can see in Table 1. Hence, while the minimum salary for the skilled non-EU/EEA migrants in 2012 was 44,800€, it increased to 46,400€ in 2013, reaching eventually the amount of 52,000€ in 2018. Similarly, the threshold of 34,944€ for employment in shortage occupations in 2012 increased to 40,560€ in 2018. Therefore, the empirical analysis will consider the time-varying thresholds.

**Table 1:** Minimum salary required for the EU Blue Card.

Year	Minimum Salary for Applicants Employed in Shortage Occupations	Minimum Salary for High Skilled Applicants
2012	34,944	44,800
2013	36,192	46,400
2014	37,128	47,600
2015	37,753	48,400
2016	38,688	49,600
2017	39,624	50,800
2018	40,560	52,000

Source: European Commission, https://ec.europa.eu/immigration/blue-card/germany_en and https://www.apply.eu/.

## Methodology

### Hypotheses and data

Three core themes have been developed to shape and explain the empirical literature on cultural participation and its role in SWB. According to the first theme, cultural participation may have a positive effect on general well-being, quality of life and physical and mental health [2, 3, 19–24]. The second theme indicates that participation in activities has little impact on the SWB [25], while the third theme argues that the effect is negligible or even statistically insignificant [25]. The differences of the outcomes and conclusions derived across the three main themes lie in assumptions about the impact of cultural participation, sampling issues, and methodological limitations [1, 26, 27].

Participation in cultural activities and its role in SWB has been mainly addressed in several contexts, communities and across various population groups, such as children, disadvantaged and marginalised groups, older adults [26]. However, there is little focus on the underrepresentation of social inequalities and the relationship among SWB, cultural participation, and social inclusion of specific groups, such as migrants. The contribution of this study relies on the fact that, to the best of our knowledge, is the first study exploring the impact of the EU Blue Card programme on migrants’ cultural participation and its impact on their SWB.

Two studies related to ours is by Schuller et al. [28] and Lin et al. [29]. Schuller et al. [28] examined the role of courses in civic integration and, in particular, language and cultural orientation. The authors evaluated the achievements in the integration process by comparing the German language proficiency between participants and non-participants. The study also looked at the role of the contacts the migrants have established with German natives. Their findings suggest that the ties with Germans increased at the end of the courses and the frequency of those contacts remained stable even 1 year after the courses. Additionally, the study concludes that by the end of the courses, the feeling of connection, attachment and belonging to Germany had also strengthened a year later. The research by Schuller et al. [28] points out that improved language skills and strong connections with German natives would have a positive impact on emotional relations and ties to Germany. Lin et al. [29] explored the relationship between social integration and health among internal migrants in ZhongShan of China. Four different measures were employed for the health status: subjective well-being, self-reported health, perception of stress, and mental health, while the social integration was measured through four dimensions: self-identity, social communication, economy, and acculturation. The results overall show that social, economic, and cultural environment can benefit migrants’ health status.

Following the discussion so far, and according to the EU Blue Card programme, the offer of employment, and the provision of a permanent residency in 2–3 years, will most likely further increase the participation in cultural activities. Thus, the first aim is to explore whether the EU Blue card programme has an impact on the participation in cultural activities for the first-generation non-EU/EEA migrants.

**H**_**1**_: EU Blue Card programme increases the cultural participation of first-generation non-EU/EEA immigrants.

The second aim is to investigate the relationship between cultural participation and SWB. This association lies in particular, in the social capital theory (SCT), which has precedents in the works of Bourdieu [30, 31], Coleman [32] and Putnam [33]. As its components have been used to investigate and describe different social phenomena, this theory is important to the purpose of our study. Previous empirical research, for example, indicates that participation in community actions has a positive effect on individual and collective well-being [34, 35], while it leads to the development of human resources; it is linked to financial and cultural capital and associated with democracy and civic participation [4, 21, 32, 34]. Hence, cultural participation improves the individuals’ social interactions, and it further enhances their SWB [3, 23]. Based on the SCT and following the evidence from the earlier studies, the second hypothesis we test is:

**H**_**2**_: Participation in cultural activities improves the SWB of both natives and immigrants.

According to earlier studies, first-generation immigrants are more likely to report lower levels of SWB due to various socio-economic and political factors, including a lower degree of social integration and embeddedness, language barriers and perceived discrimination in the labour market [36, 37]. However, even though first-generation immigrants may face various constraints, employment opportunities and labour market integration promoted by the EU Blue card programme, may also encourage the engagement in cultural events and activities, bringing people closer with those who have common cultural and social values, and also with people having different perceptions and cultural values. While it may not always be the case, these interactions and communication styles can reduce bias, prejudgment and discrimination, engender tolerance, empathy, and appreciation for differences through shared experiences, which can eventually influence positively the SWB. The next hypothesis tested is:

**H**_**3**_: Due to participation in cultural activities, the SWB levels of the treated group (first-generation non-EU/EEA migrants) will improve compared to the control group (natives and EU-migrants).

Thus, while hypothesis H_2_ tests whether the participation in cultural activities improves the SWB of both natives, EU and non-EU migrants; hypothesis H_3_ tests whether the treated group—first-generation non-EU/EEA migrants- through the participation in cultural activities, experience higher changes in the SWB compared to the control group.

### Data

This study uses anonymized secondary data, collected by the German Institute for Economic Research (DIW). The empirical analysis is based on data derived from the German Socio-Economic Panel (GSOEP), a large and nationally representative longitudinal dataset with a sample size of around 11,000 private households and more than 25,000 individuals. The survey was launched in 1984 in West Germany and it was then expanded to include the whole of Germany in 1990. The survey ethics are monitored by an independent advisory board at the DIW, and the GSOEP data are available free of charge as scientific use files. The survey records detailed and rich information on individual and household characteristics, including key demographic, education, health, income and labour market characteristics among others (For additional description and more details see https://www.diw.de/en/soepp). According to the migration policy explored- the EU Blue Card programme- and the empirical design, we will focus on the period 2015–2018. In particular, we aim to consider the high-skilled first-generation non-EU/EEA immigrants who migrated to Germany and benefited from the EU Blue Card programme. Hence, even though the GSOEP collects annual data for around 25,000 individuals, the number of participants, and therefore, of observations will be substantially lower given the limited period of analysis. Furthermore, a percentage of the respondents are aged between 0–15 years, but the questions and information about SWB and participation in cultural activities are not collected. More specifically, 25 percent of the respondents are aged between 0 and 15 years, and thus, they are excluded from the analysis for two primary reasons. First, there is no information recorded on cultural participation. Second, they do not belong in the labour force based on the legislation and the wage is not recorded, therefore, this sample cannot be considered in the identification strategy. Additionally, almost 64 percent of the remaining sample belongs in the labour force, where almost 57 percent are employed, and 7 percent are unemployed. The rest of the 36 percent are either retired, disabled and unable to work, students, housekeepers, and on maternity leave.

We should highlight that while the GSOEP is a panel survey, meaning that we follow the same individual across time, our data over the period explored are unbalanced, implying that new samples have entered the survey. More specifically, five enlargement samples of immigrants have entered the survey, the first in 1995–2010; the second in 2011–2013 and third and fourth sample in 2013–2015 and the fifth in 2016 [38]. However, we limit the analysis in the period 2015–2018, because the programme introduced in August 2012, and also, because there is no available information about the cultural participation recorded in the period 2012–2014 and 2016. Therefore, based on the information recorded and according to the methodology described in the next sections, and in particular, for the sharp regression discontinuity design (RDD) we will limit our analysis in the years 2015 and 2017–2018. However, for the fuzzy RDD and the SURE regressions presented in the Results section, we limit our empirical analysis only to 2018, since no related information on whether the respondents are granted the EU Blue card is available in the years before. We should highlight that in the case of the sharp RDD we have an intention-to-treat (ITT) analysis, which we discuss as part of the limitations of this study in the Results section.

In Table 2 we report the frequency of participation in the cultural activities explored across four groups; German natives, EU migrants, and non-EU migrants. We observe that 5.58 percent of the German natives attend cinema and jazz-pop concerts weekly; the respective percentage for the EU migrants and EU Blue card non-EU migrants are respectively 3.50 and 3.59, which are very similar. The differences are also confirmed by the Kruskal-Wallis test, which is used to test the differences in means of categorical variables between two groups, and it follows the Chi-square distribution. Thus, in the first case, we compare the German natives versus the non-EU migrants, and the high value of Kruskal-Wallis test (KS = 3,876.654) and is associated *p-value* = 0.000, shows that German natives participate more frequently than non-EU migrants. Similarly, the value of Kruskal-Wallis test of 280.532 and the *p-value* = 0.000 shows the differences in participation between EU and non-EU migrants.

**Table 2:** Frequency of participation.

**Attendance to cinema, jazz and pop concerts**	**German Natives**	**EU Migrants**	**Non-EU Migrants**	**Kruskal-Wallis chi-square test**	**Kruskal-Wallis chi-square test**
				**German natives versus non-EU migrants**	**EU Migrants versus non-EU migrants**
Every Week	5.58	3.50	2.90	7,828.791 [0.000]	280.532 [0.000]
Every Month	15.82	10.82	8.65		
Rare	39.90	29.38	24.00		
Never	38.70	56.30	64.45		
**Panel B: Attendance to Classical Music Performance, Opera or Theatre**	**German Natives**	**EU Migrants**	**Non-EU Migrants**	**Kruskal-Wallis chi-square test**	**Kruskal-Wallis chi-square test**
				**German natives versus non-EU migrants**	**EU Migrants versus non-EU migrants**
Every Week	3.17	8.02	5.46	3,876.654 [0.000]	139.142 [0.000]
Every Month	15.23	10.43	7.99		
Rare	52.96	33.45	33.59		
Never	28.64	48.10	52.96		
**Panel C: Practising Artistic Activities**	**German Natives**	**EU Migrants**	**Non-EU Migrants**	**Kruskal-Wallis chi-square test**	**Kruskal-Wallis chi-square test**
				**German natives versus non-EU migrants**	**EU Migrants versus non-EU migrants**
Every Day	1.23	0.95	1.52	3,035.888 [0.000]	85.648 [0.000]
Every Week	12.32	10.18	7.22		
Every Month	8.64	7.82	6.34		
Rare	27.57	22.60	18.40		
Never	50.24	58.45	66.52		

p-Values within the brackets.

Similar concluding remarks are derived from the proportions of participation frequency in the activities of attendance to classical music performance, opera or theatre, and practising artistic activities. However, the average frequency does not reveal any relationship, thus, we will discuss in the next section the identification strategies followed in this study. Moreover, the main objective of this study is to explore whether the EU Blue card programme has a positive impact on participation in cultural activities and whether they experience higher positive changes in their SWB, because of the programme, compared to the German natives and EU migrants who are not eligible to participate in the particular programme.

In Table 3 we report the summary statistics of life satisfaction and the main demographic and economic characteristics, such as gender, age and labour income. Interestingly, both EU and non-EU migrants report higher levels of life satisfaction, which is also confirmed by the *t-statistic* test and the *p-values*. For instance, the *t-statistic* for the average difference in life satisfaction between German natives and non-EU migrants is -3.365, with a zero p-value. On the contrary, we find no difference in the average life satisfaction levels between EU and non-EU migrants (t-statistic = 0.0537, p-value = 0.9572). While there is no clear explanation about the difference in the life satisfaction between natives and migrants, and since it is out of the current study’s objective, we will not further explore it. Nevertheless, according to the previous literature, people who have accomplished more in their lives, including education, academic standards, and career prospects experience lower positive changes in their SWB with further improvements on those standards. Furthermore, migrants and especially economic and labour migrants, and refugees present significantly higher positive changes in their life satisfaction with small improvements in their accomplishments and quality of life, such as finding a job, studying at the university, being accepted and feeling welcomed in the host society, or getting a promotion [39, 40].

**Table 3:** Summary statistics.

**Ordered and Continuous variables**	**Average**	**Standard Deviation**	**Minimum**	**Maximum**
**Life Satisfaction**				
German Natives	7.309	1.694	0	10
EU Migrants	7.470	1.699	0	10
Non-EU Migrants	7.468	1.847	0	10
	**German natives versus non-EU migrants**	**EU Migrants versus non-EU migrants**		
T-statistic test	-3.365 [0.000]	0.0537 [0.9572]	-7.146 [0.000]	
**Age**	**Average**	**Standard Deviation**	**Minimum**	**Maximum**
German Natives	43.359	12.398	16	68
EU Migrants	42.862	12.051	16	71
Non-EU Migrants	40.697	11.929	16	69
T-statistic test	22.627 [0.000]	15.474 [0.000]		
	**German natives versus non-EU migrants**	**EU Migrants versus non-EU migrants**		
**Annual Labour Income**	**Average**	**Standard Deviation**	**Minimum**	**Maximum**
German Natives	59,810.16	49,048.23	202	1,918,034
EU Migrants	49,673.65	42,039.98	121	900,500
Non-EU Migrants	36,350.88	31,957.38	108	360,000
	**German natives versus non-EU migrants**	**EU Migrants versus non-EU migrants**		
T-statistic test	47.885 [0.000]	27.036 [0.000]		
**Gender**	**Males**	**Females**	**Respondents’ population by Native-Migrant Groups in the period 2015, and 2017–2018**	**Sample Size (Percentage)**
German Natives	48.43	51.57	German Natives	41,182 (0.745)
EU Migrants	46.46	53.54	EU Migrants	4,291 (0.08)
Non-EU Migrants	51.19	48.81	Non-EU Migrants	9,775 (0.175)
**Respondents’ Population by Native-Migrant Groups in 2018**	**Sample Size (Percentage)**	**Size of Respondents by Native-Migrant Groups in the period 2015, and 2017–2018 and by Eligibility Status**	**Sample Size (Percentage)**	
German Natives	16,276 (0.73)	German Natives	10,775 (0.74)	
EU Migrants	2,051 (0.09)	EU Migrants	1,193 (0.08)	
Non-EU Migrants	5,032 (0.23)	Non-EU Migrants	2,547 (0.18)	
**Size of Respondents by Native-Migrant Groups in the period 2015, and 2017–2018 by Eligibility Status and a Bandwidth of** €**3,000**	**Sample Size (Percentage)**	**Size of Respondents by Native-Migrant Groups in the period 2015, and 2017–2018 by Eligibility Status and a Bandwidth of** €**4,000**	**Sample Size (Percentage)**	
German Natives	881 (0.72)	German Natives	1,179 (0.71)	
EU Migrants	133 (0.11)	EU Migrants	201 (0.12)	
Non-EU Migrants	204 (0.17)	Non-EU Migrants	278 (0.17)	

p-Values within the brackets.

The next variable is the annual labour income, which is one of the main criteria determining the eligibility to the EU Blue Card programme as we have discussed in the second section. We observe large income inequalities, as the average labour income for the natives’ sample is around 59,800€, significantly higher than the average income of the non-EU immigrants at 36,350€. On the contrary, the average labour income of the EU migrants is roughly 49,600€, but still lower compared to the income German natives earn on average. The average age of German natives is around 43.4 which is slightly higher than EU migrants’ average age at 42.862, while the average age of non-EU migrants is 40.7. Regarding gender, we find significant differences, as 48.43 percent of the German natives are males and the remaining 51.57 percent are females, while the respective values for the EU migrants are 46.46 and 53.54 percent, and for the non-EU migrants are 51.19 and 48.81 percent.

Next, we report the sample size and the proportions of the natives and migrants included in the empirical analysis. In particular, the sample size of the German Natives in the period 2015 and 2017–2018 is 41,182 comprising 74.5 percent of the total sample, followed by 9,775 non-EU migrants at 17.5 percent and 4,291 EU migrants at 8 percent. However, the sample used in this period is further limited to those who are eligible. More specifically, following the discussion in the second section about the eligibility criteria of the EU Blue Card programme, we limit the sample to those who are employed in shortage occupations or are high-skilled workers that we discuss in the next section. In this case, the sample size for the German natives becomes 10,775 at 74 percent; 2,547 for the non-EU migrants at 18 percent, and 1,193 for the EU migrants at 8 percent. These statistics are representative, as are very similar to the migrant statistics of EUROSTAT (For more information see, https://ec.europa.eu/eurostat/statistics-explained/index.php/Migration_and_migrant_population_statistics).

Then we further limit the sample within the bandwidth of 2,000€; 3,000€, and 4,000€ and using the cutoff points or thresholds reported in Table 1, to implement the RDD that we describe in more details in the next section. Thus, for instance, in 2015, those who are employed in shortage occupations and considering a bandwidth of 3,000€ we obtain those who earn between 34,753€ and 40,753€ based on a threshold value of a labour income of 37,753€ using the statistics in Table 1. Additionally, based on Table 1, for the same bandwidth, we consider the high-skilled workers earning between 45,400–51,400 given a threshold value of 48,400€. Therefore, the sample size includes the sum of both high-skilled workers and those employed in shortage occupations. We report the sample size considering the bandwidths of 3,000€, and 4,000€. For a bandwidth of 3,000€, the sample of the empirical analysis in the sharp RDD includes 881 German natives; 133 EU migrants, and 204 non-EU migrants respectively at 72, 11 and 17 percent. Similarly, using the bandwidth of and 4,000€, 1,179 German natives at 71 percent, 201 EU migrants at 12 percent and 278 non-EU migrants at 17 percent.

Thus, overall, we conclude that the sample size is reduced by removing those who are aged between 0 and 15 years and do not belong in the labour force, while we consider only those who are high-skilled workers or employed in shortage occupations. To further infer causality we employ three bandwidths and apply the sharp RDD as we discuss in more details in the next section. Finally, we report the sample size across native and migrant groups in 2018. This sample will be used in the ordered Probit SURE and the Fuzzy RDD since both methods require information about whether the respondents are granted the EU Blue Card. Since this information is recorded only in 2018, we will limit our analysis to that year. The sample includes 16,276 German natives comprising 73 percent of the sample; 2,051 EU migrants at 9 percent, and 5,032 non-EU migrants at 23 percent. We should highlight that the sample, in this case, is larger than the one described in the case of the total sample over the years 2015, and 2017–2018, because in the former sample we include all the natives and migrants irrespective of the eligibility, while, in the latter case we limit the sample only to those employed in shortage occupations and the high-skilled workers, according to the EU Blue Card programme eligibility criteria. Thus, we use the full sample in the former case and we apply the Ordered Probit simultaneous regressions and additionally, we implement the fuzzy RDD, which is an instrumental variables (IV) approach. In this case, we apply the Two-Stage Least Squares (2SLS) method and we instrument the participation in the EU Blue Card programme with the eligibility criteria, described in more details in the next section.

### Sharp regression discontinuity design (RDD)

The objective of this section is to present the sharp and fuzzy RDD within a seemingly unrelated regression equations (SURE) system to explore the impact of the EU Blue Card programme on non-EU/EEA migrants’ cultural participation, and to investigate the impact of cultural participation on the SWB. The main justification and underlying reason for proposing an RDD framework is to infer causality from our results. In particular, while cultural participation may improve the SWB, also the latter may influence the participation in cultural activities. Furthermore, participation in cultural and recreational activities, as part of leisure time, are unlikely to be independent of employment decisions. Therefore, to identify the impact of the EU Blue Card programme on cultural participation and the impact of the latter on SWB, we exploit the exogenous variation in the employment offer close to the thresholds of the salaries reported in Table 1.

The RDD approach has several advantages discussed in earlier studies [41, 42], essentially, because individuals are close to the cut-off point or on the two sides of the threshold of 44,800€ for those who have completed a higher education degree and 34,944€ for those who are employed in specific professions. As we have highlighted, these thresholds refer to the year 2012, while for the period 2015–2018 we will consider the minimum salaries reported in Table 1 as thresholds or cut-off points.

RDD is the closest to a randomised controlled trial (RCT) that can be used in non-experimental settings, such as this study relies on the GSOEP. Additionally, this strategy requires fewer assumptions, compared to other methods, with the differences-in-differences (DID) framework being the most prevalent method, and which is focusing on finding a control group that is very similar to the treatment group. The first identification strategy relies on a sharp RDD using the two thresholds mentioned earlier.


$$CP_{ijt}=\beta_{0}D_{ijt}+\beta_{1}f(wage_{ijt}-c)+\beta_{2}D_{ijt}*f(wage_{ijt}-c)+u_{ijt}^{}$$ (1)

$$SWB_{ijt}=\gamma_{0}D_{ijt}+\gamma_{1}f(wage_{ijt}-c)+\gamma_{2}D_{ijt}*f(wage_{ijt}-c)+\gamma_{3}CP_{ijt}+\gamma_{4}D_{ijt}*CP_{ijt}+e_{ijt}^{}$$ (2)

In the structural equation system 1)-(2), *CP* is the cultural participation for individual *i*, in area-state *j*, measured by the Nomenclature of Territorial Units for Statistics (NUTS) 1 level, and time *t*. Thus, the empirical analysis relies on panel data implying that we follow the same individual across the period we examine and is 2015–2018. *SWB* denotes subjective well-being which is life satisfaction and is measured on an 11 point Likert scale between 0-indicating complete dissatisfaction- and 10- denoting complete satisfaction. The variables expressing the participation in cultural activities are Likert variables measured on a scale from 1 (weekly) to 4 (never), thus, a positive coefficient would imply a lower frequency in cultural participation. Nonetheless, to avoid confusion and to simplify the interpretation of the estimates, we have recoded the *CP* outcomes in such a way that a higher value shows a higher frequency, and in particular, 1 denotes *never* and 4 shows *weekly* participation. We explore three cultural activities: participation in cinema and jazz/pop concerts; attendance to classical music performance, opera or theatre; and practising artistic activities, such as playing a musical instrument, painting-drawing or composing music among others.

The main estimated coefficient of interest is that of dummy variable *D*, indicating whether individuals are below (*D*_*i*_ = 0) or above (*D*_*i*_ = 1) the thresholds of the minimum salaries in Table 1. Thus, according to the discussion preceded about the EU blue card programme, the treated subjects in our case are non-EU/EEA first-generation immigrants who are placed above the threshold, while the control group consists of natives and both EU and non-EU/EEA immigrants who earn a gross salary lower than the minimum salaries in Table 1. Therefore, the dummy variable *D* takes a value of 1 whether the wage is above the minimum salaries in Table 1, which are the thresholds or cut-off points in our RDD specification. The analysis will take place separately for each wage threshold using three bandwidths, 2,000€; 3,000€ and 4,000€. In particular, when the threshold is above the minimum salaries required for the EU Blue Card programme reported in the first column we consider only those who are employed in specific professional classes expressed by the 4-digit International Standard Classification of Occupations (ISCO-88) for both treated and control groups, while in the second column we consider only the university graduates (https://ec.europa.eu/eurostat/documents/1978984/6037342/ISCO-88-COM.pdf). According to the EU Blue card programme and the ISCO-88, we consider professionals and technicians and associate professionals, such as physical, mathematical, engineering, life, health science professionals, teaching professionals, computer associate professionals, optical and electronic equipment operators among others.

Term *f*(*wage*_*ijt*_ − *c*) refers to the functional form of the forcing variable *wage*, which is the gross salary-wage of the respondent centered by the threshold *c*, which corresponds to the amounts of salaries in Table 1. We will estimate the system of Eqs (1) and (2) simultaneously, by applying a SURE system with the Ordered Probit method, since the outcomes in both equations are ordered-Likert variables. Eq (1) is employed to explore the causal impact of the EU Blue Card programme on the cultural participation of treated subjects (non-EU/EEA first-generation immigrants). Then, in Eq (2) we explore, not only the impact of the EU Blue Card programme on the *SWB*, expressed by the coefficient *γ*_*0*_, but we also consider the impact of cultural participation on the *SWB* denoted by the coefficient *γ*_*4*_. We should notice that we have considered also a quadratic term in the forcing variable *f*(*wage*_*ijt*_—*c*) and its interaction with *D*, and the main concluding remarks remain similar, while higher-order polynomials, e.g. cubic, become insignificant. Furthermore, the results remain robust when we include covariates, such as age, marital status, household size and income, and fixed effects for NUTS 1 and time, but we do not report the results.

### Fuzzy regression discontinuity design (FRDD)

The eligibility conditions determine that first-generation non-EU/EEA immigrants need to be employed in a job offering of gross salaries reported in Table 1, and the definitions of those thresholds may strongly influence the likelihood of a foreigner finding a job in the destination country. A high level of minimum salary, as well as higher education qualifications required for the eligibility to the EU Blue Card programme, are more likely to attract migrants who already participate more frequently in cultural activities, and are more satisfied with their lives. Furthermore, they may demonstrate a higher level of the host country’s language proficiency, higher adaptation levels to the host country’s cultural and social norms and thus, more likely to participate in the cultural activities we explore. Cultural participation signifies the social identity and status [30], while the key finding of the literature is that those belonging in the higher social class positions- defined by occupations, education and income- are more likely to participate in cultural activities [43]. Hence, if we include in our regressions only a dummy taking a value of 1 for the EU Blue Card holders and 0 otherwise, it is very likely we introduce a sample selection bias, even though we can control for income.

Moreover, apart from the minimum salary required to be eligible for the EU Blue Card programme, the scheme also requires specific professions such as engineers, physicians and computer scientists. Thus, in this context, since high salaries are indicators of labour shortages in specific job positions or sectors, a salary threshold distinguishes applicants in the sectors needed from applicants in less needed ones, hence, a positive selection is being carried out. Thus, we may include a selection bias in our estimates, by considering in the control group those who do not belong in the eligible sectors and potentially earn a significantly lower salary than the threshold required to acquire the EU Blue Card. As we have highlighted earlier, large differences in the education and income level may also explain large differences in cultural participation.

In FRDD the propensity score function Pr (*D*_*i*_ = 1 | *X*_*i*_) is discontinuous, as in the case of the sharp RDD, where *X*_*i*_ denotes the assignment variable *wage* in our case, but the difference is that instead of a 0–1 step function, the treatment probability as a function of how the *wage* now contains a jump at the cut-off point that is less than 1. In particular, the probability of those above the cut-off point is not necessarily 1, because participation in the EU Blue Card programme is not mandatory, as it is assumed in the sharp RDD framework. In other words, the eligibility to the programme is an employment offer of a salary higher than the minimum salaries presented in Table 1, but it does not imply that all the migrants are granted the EU Blue Card, because of the reasons discussed earlier. Thus, the assignment rule defines the treatment status probabilistically, between 0 and 1, but not perfectly, as it is assumed in the sharp RDD. In this case, the assignment variable *wage* is highly correlated with the treatment and it is used as an instrumental variable for the participation in the EU Blue Card programme. Therefore, implementing the FRDD, we apply in some manner a propensity score matching process, where we attempt to correct for the selection bias by including in the regression an observed variable that controls for sample selection, which is the dummy indicating whether the *wage* is above or below the cut-off point.

In this setting, the fuzzy RDD can be more qualified in the sense that the probability of job offer does not jump from 0 to 1 for workers with earnings more than the thresholds mentioned. Furthermore, the fuzzy RDD is a more suitable approach to include also those who were not eligible and have not received the EU Blue Card. Most importantly, another benefit of the fuzzy RDD is that we do not have to limit our sample within a certain bandwidth, as within the bandwidth of -4,000€ and 4,000€ we have described earlier in the sharp RDD. Therefore, we can consider the full sample of the respondents, including both those who have received the EU Blue card and those who have not. Analytically, the estimation of the treatment effect in a fuzzy RDD is often carried out by the two-stage least squares (2SLS) method. Nevertheless, since we have a system of equations to be estimated simultaneously, we will apply the Ordered Probit method. The system of equations to be estimated is:

$${Τ}_{jit}=b_{1}D_{ijt}+f(wage_{ijt}-c,a)+u_{ijt}^{}$$ (3)

$$CP_{ijt}=\beta_{0}\hat{T}{\;}_{ijt}+\beta_{1}f(wage_{ijt}-c)+\beta_{2}\hat{T}{\;}_{ijt}*f(wage_{ijt}-c)+\beta \prime \text{Z}+v_{ijt}^{}$$ (4)

$$SWB_{ijt}=\gamma_{0}\hat{T}{\;}_{ijt}+\gamma_{1}f(wage_{ijt}-c)+\gamma_{2}\hat{T}{\;}_{ijt}*f(wage_{ijt}-c)+\gamma_{3}CP_{ijt}+\gamma_{4}\hat{T}{\;}_{ijt}*CP_{ijt}+\gamma \prime \text{X}+e_{ijt}^{}$$ (5)

Where the variables are defined as in Eqs (1)–(2), except for the variable *T*, which is the actual participation in the EU Blue Card programme taking a value of 1 if the respondent has received the EU Blue Card and 0 otherwise. In particular, Eq (3) is the first stage regression where we regress the participation variable *T* on the variable *D*. Then in the next stage, the fitted values of variable *T* are included in the structural Eqs (4)–(5). Vectors **Z** and **X** include socio-economic characteristics, such as age, marital status, and professional class among others. We should notice that when we estimate the FRDD, we consider the respondents who have either completed a university education degree and higher or are employed in the specific professions mentioned earlier. However, it is important to highlight that due to data availability, and the information recorded in the GSOEP; we limit the FRDD estimates in the year 2018 since there is no relevant information or question asked about whether the respondent has been granted or not with an EU Blue Card programme in the years before 2018. Thus, the main justification for implementing additionally the sharp RDD in the years 2015 and 2017–2018 lies in the absence of information about the EU Blue Card and allowing us to exploit the panel data structure of the GSOEP.

## Results

### Main findings

In Table 4 we report the RDD estimates, using a uniform Kernel smoothing, for the participation in cinema and jazz/pop concerts. In panel A, we report the sharp RDD when the cut-off point or threshold corresponds to the minimum salaries required for the EU Blue Card programme and those employed in shortage occupations, as we have shown in Table 1, while in panel B we present the results for the threshold of high-skilled occupations. We observe that for different bandwidth levels, the results confirm hypothesis H_2_, where attendance to cinema and jazz/pop concerts contributes positively to life satisfaction for natives and migrants.

**Table 4:** RDD seemingly unrelated regression estimates using Ordered Probit and uniform Kernel for participation in cinema and jazz/pop concerts.

**Panel A: Employed in Shortage Occupations**	Bandwidth 2,000€	Bandwidth 3,000€	Bandwidth 4,000€
**First Regression: Cultural Participation**	DV: Participation	DV: Participation	DV: Participation
β_0_ (Treat)	0.2828 (0.2545)	0.3388* (0.1775)	0.3371* (0.1812)
β_1_(wage − c)	0.0003 (0.0007)	0.0003* (0.00016)	0.0003 (0.0002)
β_2_(Treat)* (wage -c)	0.0002* (0.00011)	0.0003** (0.00014)	0.00035** (0.00016)
**Second Regression: SWB**	DV: Life Satisfaction	DV: Life Satisfaction	DV: Life Satisfaction
γ_0_ (Treat)	0.7609 (0.6630)	0.8647 (0.6561)	0.7461 (0.6954)
γ_3_(CP)	0.3456** (0.1522)	0.3828*** (0.1014)	0.3609** (0.1422)
γ_4_(Treat)* (CP)	0.2434 (0.1775)	0.2621 (0.1838)	0.2518 (0.2519)
No. observations	478	699	953
Log-Likelihood	-1,420.87	-2,111.26	-2,857.62
**Panel B: High Skilled**	Bandwidth 2,000€	Bandwidth 3,000€	Bandwidth 4,000€
**First Regression: Cultural Participation**	DV: Participation	DV: Participation	DV: Participation
β_0_ (Treat)	0.2591** (0.1113)	0.2925** (0.1316)	0.2563** (0.1222)
β_1_ (wage-c)	0.0003** (0.00013)	0.00028** (0.00013)	0.00079* (0.00041)
β_2_(Treat)* (wage − c)	0.0004** (0.0002)	0.0003** (0.00014)	0.0017** (0.0008)
**Second Regression: SWB**	DV: Life Satisfaction	DV: Life Satisfaction	DV: Life Satisfaction
γ_0_ (Treat)	1.4218** (0.5828)	1.2869** (0.5179)	1.3336** (0.5983)
γ_3_(CP)	0.3365** (0.1563)	0.3342** (0.1364)	0.4229*** (0.1352)
γ_4_(Treat)* (CP)	0.1912** (0.0893)	0.1794* (0.1052)	0.1823** (0.0815)
No. observations	324	519	665
Log-Likelihood	-926.87	-1,516.38	-1,942.47

Robust standard errors within parentheses,

***, ** and * indicate significance at 1%, 5% and 10% level. DV denotes dependent variable and CP stands for cultural participation.

Based on panel A, the main coefficient of interest *β*_*0*_ (Treat) is insignificant, implying that the EU Blue card programme does not increase the cultural participation of the treated subjects compared to the control group when the bandwidth is 2,000€; however, it becomes significant in the RDD estimates using the bandwidths of 3,000€ and 4,000€, confirming hypothesis H_1_. However, the EU Blue Card programme does not affect well-being, as we see the insignificant estimated coefficients in the second regression of the sharp RDD system (1)-(2), which does not confirm hypothesis H_3_. On the other hand, the results for the high-skilled positions, show a positive impact of the EU Blue Card programme on participants’ life satisfaction. Furthermore, according to the coefficient *γ*_*4*_ of the interaction term Treat* *CP* in regression (2), we accept hypothesis H_3_, implying that even though both treated and control groups improve their SWB based on the coefficient *γ*_*3*_, participation in cinema and jazz-pop concerts contribute to higher positive changes in life satisfaction of the treated group which comprises first-generation non-EU/EEA migrants, compared to the control group that consists of natives and EU-migrants.

The same concluding remarks are derived when we explore the participation in classical music performance, opera or theatre in Table 5 and practising artistic activities in Table 6. In particular, in the first regression, we find a significant impact of the EU Blue Card programme on the participation of non-EU migrants employed in shortage occupations, confirming hypothesis H_1_, but we find no impact on the SWB and insignificant differences in the changes of life satisfaction between treated and control groups. Regarding practising artistic activities, the results in Table 6 show that the EU Blue Card programme does not affect the non-EU migrants’ participation, and in particular, those who are employed in shortage occupations. Additionally, the results in the second regression do not confirm hypothesis H_3_, implying the life satisfaction of the non-EU migrants is not improved more, because of practising artistic activities, compared to the SWB of the control groups, even though the results show an improvement in the SWB of non-EU migrants, according to the coefficient *γ*_*0*._

**Table 5:** RDD seemingly unrelated regression estimates using Ordered Probit and uniform Kernel for participation in classical music performance, opera or theatre.

**Panel A: Employed in Shortage Occupations**	Bandwidth 2,000€	Bandwidth 3,000€	Bandwidth 4,000€
**First Regression: Cultural Participation**	DV: Participation	DV: Participation	DV: Participation
β_0_ (Treat)	0.2421 (0.1620)	0.2449* (0.1342)	0.2319* (0.1294)
β_1_ (wage − c)	-0.0041 (0.0062)	0.0069* (0.0039)	0.0067** (0.0033)
β_2_(Treat)* (wage − c)	0.0015 (0.0021)	0.0016 (0.0012)	0.0019 (0.0021)
**Second Regression: SWB**	DV: Life Satisfaction	DV: Life Satisfaction	DV: Life Satisfaction
γ_0_ (Treat)	1.2846 (1.1026)	1.5516 (1.3770)	1.5641 (1.3177)
γ_3_(CP)	0.1763** (0.0771)	0.1854*** (0.0465)	0.1787*** (0.0401)
γ_4_(Treat)* (CP)	0.1988 (0.3074)	0.2435 (0.2672)	0.2070 (0.1664)
No. observations	478	699	953
Log-Likelihood	-1,424.86	-2,106.51	-2,668.82
**Panel B: High Skilled**	Bandwidth 2,000€	Bandwidth 3,000€	Bandwidth 4,000€
**First Regression: Cultural Participation**	DV: Participation	DV: Participation	DV: Participation
β_0_ (Treat)	0.1623 (0.2227)	0.3106* (0.1798)	0.2630** (0.1221)
β_1_(wage -c)	0.0048* (0.0025)	0.0064* (0.0033)	0.0065** (0.0032)
β_2_(Treat)* (wage − c)	0.0028 (0.0032)	0.0048 (0.0117)	0.0045 (0.0112)
**Second Regression: SWB**	DV: Life Satisfaction	DV: Life Satisfaction	DV: Life Satisfaction
γ_0_ (Treat)	1.5694* (0.8887)	1.7282** (0.8058)	1.7529*** (0.5734)
γ_3_(CP)	0.4848** (0.2276)	0.4730*** (0.2274)	0.4522** (0.2265)
γ_4_(Treat)* (CP)	0.4095* (0.2278)	0.4093* (0.2258)	0.4097** (0.1614)
No. observations	324	519	665
Log-Likelihood	-869.65	-1,491.61	-1,955.57

Robust standard errors within parentheses,

***, ** and * indicate significance at 1%, 5% and 10% level. DV denotes dependent variable and CP stands for cultural participation.

**Table 6:** RDD seemingly unrelated regression estimates using Ordered Probit and uniform Kernel for participation in practising artistic activities.

**Panel A: Employed in Shortage Occupations**	Bandwidth 2,000€	Bandwidth 3,000€	Bandwidth 4,000€
**First Regression: Cultural Participation**	DV: Participation	DV: Participation	DV: Participation
β_0_ (Treat)	0.2465 (0.3584)	0.2268 (0.2414)	0.2914 (0.2554)
β_1_(wage − c)	0.0025 (0.0029)	0.0021 (0.0020)	0.0022 (0.0018)
β_2_(Treat)* (wage -c)	0.0025 (0.0033)	0.0020 (0.0018)	0.0022 (0.0015)
**Second Regression: SWB**	DV: Life Satisfaction	DV: Life Satisfaction	DV: Life Satisfaction
γ_0_ (Treat)	0.9787 (0.8171)	1.0316** (0.5162)	0.9771* (0.4345)
γ_3_(CP)	0.1509* (0.0783)	0.1863** (0.0804)	0.1427** (0.0681)
γ_4_(Treat)* (CP)	0.1563 (0.1262)	0.1233 (0.1078)	0.1660 (0.1939)
No. observations	478	699	953
Log-Likelihood	-1,413.45	-2,096.58	-2,846.84
**Panel B: High Skilled**	Bandwidth 2,000€	Bandwidth 3,000€	Bandwidth 4,000€
**First Regression: Cultural Participation**	DV: Participation	DV: Participation	DV: Participation
β_0_ (Treat)	0.4996* (0.2662)	0.5101** (0.2424)	0.4997** (0.2451)
β_1_(wage -c)	0.0031** (0.0015)	0.0028** (0.0014)	0.0028** (0.0012)
β_2_(Treat)* (wage -c)	0.0007 (0.0035)	0.0022 (0.0019)	0.0019 (0.0013)
**Second Regression: SWB**	DV: Life Satisfaction	DV: Life Satisfaction	DV: Life Satisfaction
γ_0_ (Treat)	0.8372 (0.5313)	0.9405* (0.5641)	0.8415* (0.4849)
γ_3_(CP)	0.1835* (0.1020)	0.1861** (0.0863)	0.1917** (0.0821)
γ_4_(Treat)* (CP)	0.1980 (0.1494)	0.2491** (0.1209)	0.2377* (0.1214)
No. observations	324	519	665
Log-Likelihood	-971.27	-1,577.15	-2,017.78

Robust standard errors within parentheses,

** and * indicate significance at 5% and 10% level. DV denotes Dependent variable and CP stands for cultural participation.

On the other hand, high-skilled non-EU/EEA first-generation immigrants who earn more than the thresholds in the second column of Table 1, are more likely to participate in all cultural activities we explore, indicated by the positive coefficient *β*_*0*_ *(Treat)* in panel B of Tables 4–6. Furthermore, we find that the EU Blue Card programme improves the SWB of the non-EU migrants more compared to the SWB of the control group, as it can be seen from the coefficient *γ*_*4*_ of the interaction term Treat*CP in regression 2, confirming hypothesis H_3_.

As a robustness check, we repeat the estimates of the sharp RDD system (1)-(2) using a triangle Kernel smoothing and a bandwidth of 4,000€. In panels A and B of Table 7 we report the estimates using respectively the samples of those employed in shortage occupations and those who are high-skilled workers. The results are very close to those reported in Tables 4–6, confirming the robustness of sharp RDD using a uniform Kernel smoothing.

**Table 7:** RDD seemingly unrelated regression estimates for cultural participation and life satisfaction using a triangle Kernel and bandwidth of 4,000€.

	Panel A: Employed in Shortage Occupations			Panel B: High Skilled		
**First Regression: Cultural Participation**	DV: Participation in Cinema and Jazz/Pop Concerts	DV: Participation in Classical Music Performance, Opera or Theatre	DV: Participation in Practising Artistic Activities	DV: Participation in Cinema and Jazz/Pop Concerts	DV: Participation in Classical Music Performance, Opera or Theatre	DV: Participation in Practising Artistic Activities
β_0_ (Treat)	0.2902* (0.1689)	0.2122** (0.1019)	0.2852 (0.2426)	0.2528** (0.1122)	0.2795** (0.1311)	0.4797** (0.2135)
β_1_(wage -c)	0.00025 (0.0004)	0.0069** (0.0032)	0.0028 (0.0021)	0.00071* (0.00038)	0.0084** (0.0039)	0.0025* (0.0013)
β_2_(Treat)* (wage − c)	0.00041* (0.00021)	0.0014 (0.0012)	0.0026 (0.0022)	0.0018** (0.0009)	0.0052 (0.0101)	0.0019 (0.0012)
**Second Regression: SWB**	DV: Life Satisfaction	DV: Life Satisfaction	DV: Life Satisfaction	DV: Life Satisfaction	DV: Life Satisfaction	DV: Life Satisfaction
γ_0_ (Treat)	0.8142 (0.8146)	1.4662 (1.2096)	0.9186** (0.4420)	1.1210*** (0.4268)	1.9064*** (0.6048)	0.9180** (0.4501)
γ_3_(CP)	0.3570*** (0.1263)	0.1648*** (0.0371)	0.1490** (0.0657)	0.3834*** (0.1219)	0.4668** (0.2144)	0.2031** (0.0982)
γ_4_(Treat)* (CP)	0.2321 (0.2022)	0.1885 (0.1769)	0.1721 (0.1568)	0.2189** (0.1035)	0.4534** (0.1876)	0.2557** (0.1202)
No. observations	953	953	953	665	665	665
Log-Likelihood	-2,864.65	-2,666.89	-2,844.76	-1,893.42	-1,816.85	-1,972.42

Robust ctandard errors within parentheses,

***, ** and * indicate significance at 1%, 5% and 10% level. DV denotes dependent variable and stands for cultural participation.

**Fig 1:**
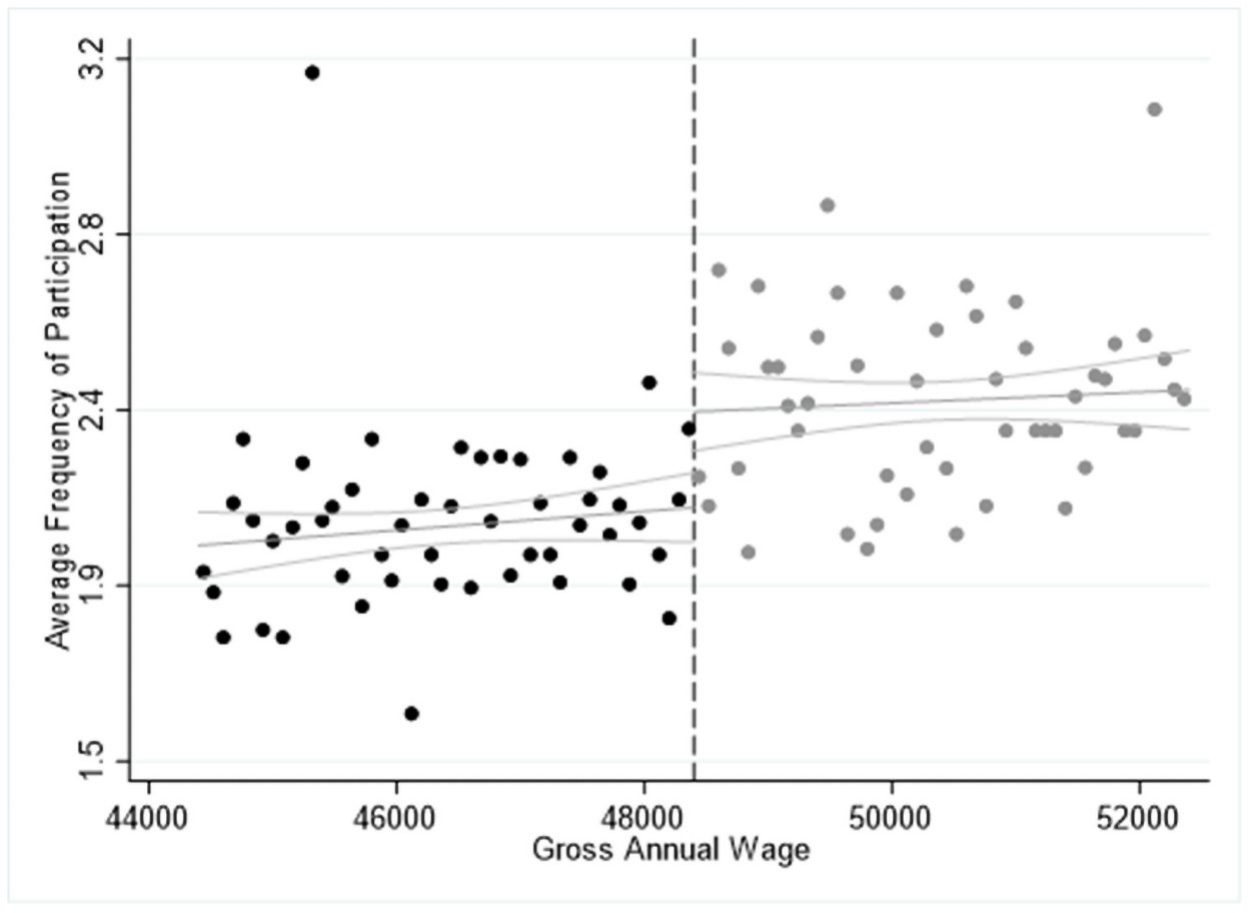
Sharp RDD estimates for participation in cinema and jazz/pop concerts using the salary threshold of 48,400€ in 2015.

**Fig 2:**
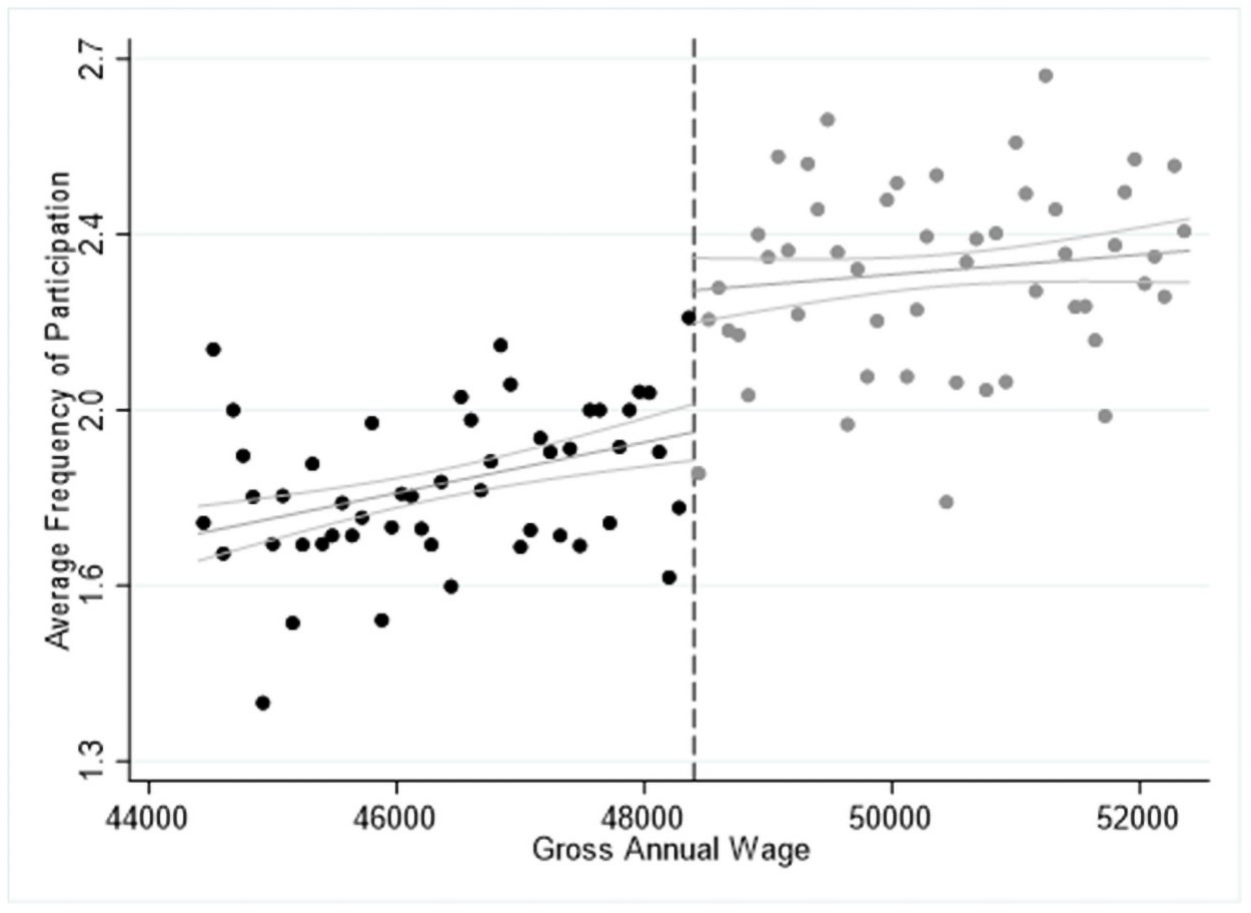
Sharp RDD estimates for participation in classical music performance, opera or theatre using the salary threshold of 48,400€ in 2015.

**Fig 3:**
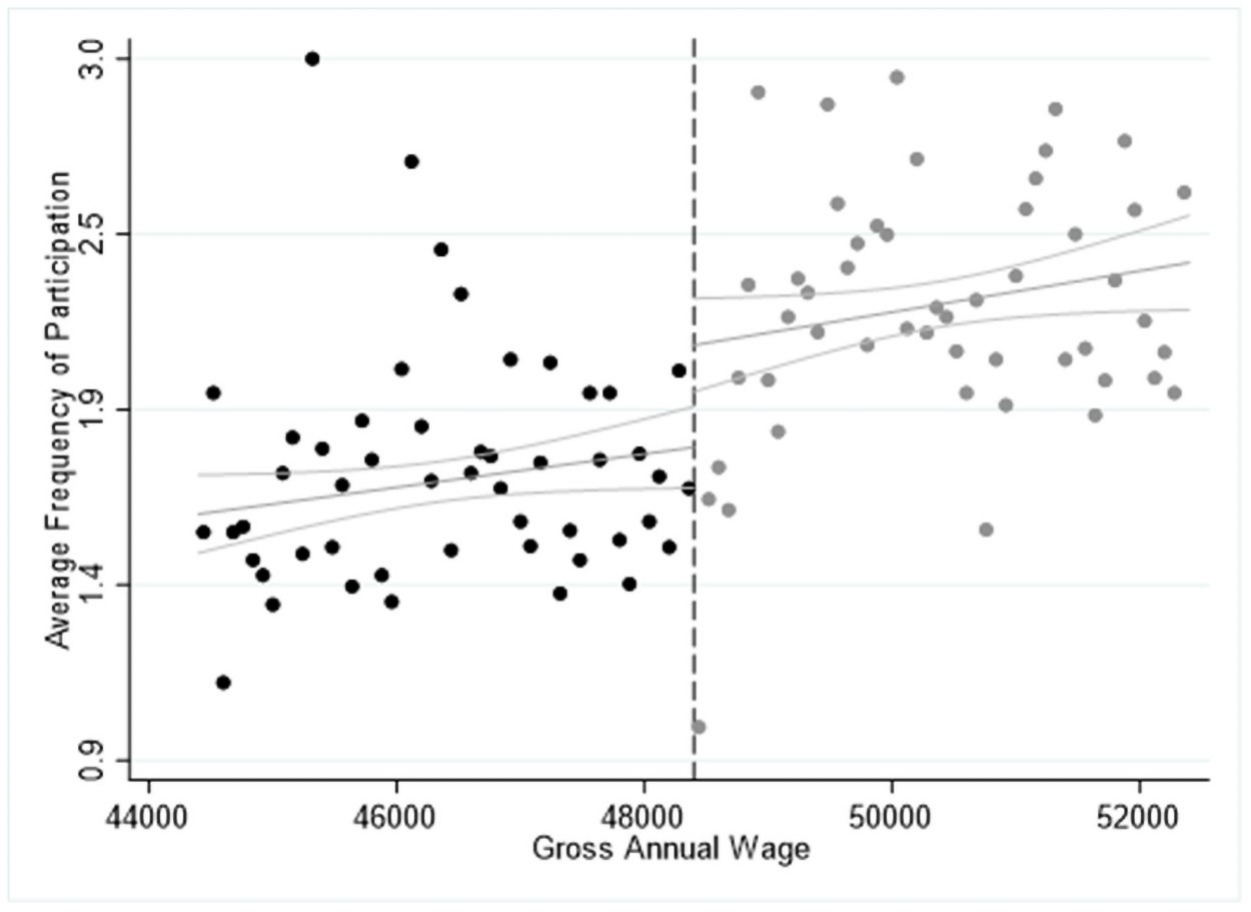
Sharp RDD estimates for participation in practising artistic activities using the salary threshold of 48,400€ in 2015.

The results in Tables 4–6 are confirmed by Figs 1–3, where we report the average frequency participation in 2015 for each activity we explore and we highlight the cut-off point of the salary for high-skilled workers at of 48,400€ by the dashed vertical line. In all cases, we observe a significant “jump” upwards in the ride side of the cut-off point showing that eligible respondents are more likely to participate more frequently in the cultural activities explored. Furthermore, Figs 1–3 seem to confirm the specification of the model, which can be approximated by a linear or a quadratic relationship while higher polynomial orders are found statistically insignificant. Moreover, the slope of the estimated curve is positive, indicating that there is a positive relationship between income and average frequency of participation in cultural activities.

In Table 8 we report the estimates derived from the SURE fuzzy regression discontinuity regression (FRDD) system (3)-(5). Overall, based on the results and the first regression (cultural participation), the EU Blue programme had a higher impact on the participation in classical music performance, theatre or opera followed by practising activities for the high-skilled migrants, followed by and participation in cinema and jazz-pop concerts. On the contrary, when we consider those employed in the shortage occupations in panel A, we observe that the programme has a higher impact on the participation in cinema and jazz-pop concerts, followed by practising activities, and participation in classical music performance, theatre or opera.

**Table 8:** FRDD estimates full sample.

	Panel A: Employed in Shortage Occupations			Panel B: High Skilled		
**First Regression: Cultural Participation**	DV: Participation in Cinema and Jazz/Pop Concerts	DV: Participation in Classical Musical Performance, Opera or Theatre	DV: Practising Artistic Activities	DV: Participation in Cinema and Jazz/Pop Concerts	DV: Participation in Classical Musical Performance, Opera or Theatre	DV: Practising Artistic Activities
β_0_ (Treat)	0.2554** (0.1211)	0.1851** (0.0825)	0.2882*** (0.0846)	0.1533** (0.0760)	0.3218*** (0.0303)	0.2832*** (0.0646)
**Second Regression: SWB**	DV: Life Satisfaction	DV: Life Satisfaction	DV: Life Satisfaction	DV: Life Satisfaction	DV: Life Satisfaction	DV: Life Satisfaction
γ_0_ (Treat)	0.2503** (0.1203)	0.1327 (0.2568)	0.3917** (0.1861)	0.8384** (0.3938)	0.9318** (0.4353)	0.6858** (0.3354)
γ_3_(CP)	0.1728*** (0.0274)	0.2823*** (0.0286)	0.0372*** (0.0178)	0.1269*** (0.0232)	0.2631*** (0.0281)	0.0484** (0.0066)
γ_4_(Treat)* (CP)	0.1388** (0.0633)	0.1034* (0.0597)	0.0728 (0.0594)	0.1001 (0.0808)	0.0897** (0.0432)	0.1071* (0.0572)
No. observations	22,359	22,359	22,359	22,359	22,359	22,359
Log-Likelihood	-16,315.43	-16,022.88	-16,569.40	-18,481.15	-16,716.59	-18,407.06
Weak Instrument F-Statistic Test	43.59 [0.000]	73.47 [0.000]	77.61 [0.000]	54.36 [0.000]	35.18 [0.000]	51.33 [0.000]
Hausman Endogeneity Test	0.0256 (0.0644)	-0.1121 (0.1959)	-0.2595 (0.1627)	-0.7171 (0.9002)	0.5156 (0.4453)	-0.6187 (0.5817)

Robust standard errors within parentheses, P-values within brackets,

***, ** and * indicate significance at 1%, 5% and 10% level. DV denotes dependent variable and CP stands for cultural participation.

EU blue card programme has a positive impact on the improvement of SWB, indicated from the coefficient *γ*_*0*_, confirming the findings derived in earlier studies, showing that engagement in arts activities has positive implications for well-being [43–46]. The beneficial effect of artistic activities and participation in cultural activities could be due to several mechanisms, such as these activities may promote self-expression and creativity; enhance self-identity by pursuing skills; build the social identity of an individual; enhance social support, and reduce psychological, biological stress markers, and depression-related sedentary behaviours [47–51].

Regarding hypothesis H_3_, we find that non-EU migrants employed in shortage occupations and participate more frequently in all cultural activities explored are more likely to experience higher positive changes in life satisfaction compared to the control group, indicated by the positive coefficient *γ*_*4*._ Therefore, while both treated and control groups improve their life satisfaction, because of attendance to the three types of cultural activities, shown by the coefficient *γ*_*3*_ in Table 8, non-EU migrants improve their life satisfaction more than the respondents belonging in the control group do.

In all cases, based on the weak instrument *F-test* and its associated *p-values*, we reject the null hypothesis, concluding that the instrument employed is not weak and is a good predictor of cultural participation. More specifically, as a rule of thumb, the *F-statistic* test should be higher than 10 and it is derived from the first stage regression (3). Furthermore, we report the Hausman endogeneity test and in all cases, we accept the joint null hypothesis implying that the instruments used are exogenous, uncorrelated with the error term and they might have an indirect effect on the SWB only through the participation in cultural activities.

In Table 9 we report the FRDD using the bandwidth of 4,000€ to check the robustness of the estimates in Table 8. The results confirm the significance of the EU Blue Card Programme on cultural participation of the first generation non-EU/EEA migrants and its impact on their life satisfaction. Furthermore, the results confirm hypothesis H_2_, where both natives and migrants participating in the cultural activities explored are more likely to report higher levels of SWB. Nevertheless, the advantage of the FRDD is that we are allowed to use the full sample, rather than limiting our sample only within a specific bandwidth, as in the case of the sharp RDD.

**Table 9:** FRDD estimates using a bandwidth of 4,000€.

	Panel A: Employed in Shortage Occupations			Panel B: High Skilled		
**First Regression: Cultural Participation**	DV: Participation in Cinema and Jazz/Pop Concerts	DV: Participation in Classical Musical Performance, Opera or Theatre	DV: Practising Artistic Activities	DV: Participation in Cinema and Jazz/Pop Concerts	DV: Participation in Classical Musical Performance, Opera or Theatre	DV: Practising Artistic Activities
β_0_ (Treat)	0.3190* (0.1789)	0.1453* (0.0622)	0.1821** (0.0793)	0.2920** (0.1389)	0.3568** (0.1645)	0.4182** (0.1960)
**Second Regression: SWB**	DV: Life Satisfaction	DV: Life Satisfaction	DV: Life Satisfaction	DV: Life Satisfaction	DV: Life Satisfaction	DV: Life Satisfaction
γ_0_ (Treat)	0.4507 (0.3966)	0.2306 (0.3238)	0.5184* (0.2863)	1.1913** (0.5815)	1.7635* (0.9753)	1.1778 (0.8949)
γ_3_(CP)	0.2467** (0.0998)	0.2610*** (0.0961)	0.0463** (0.0224)	0.1203** (0.0523)	0.2217** (0.0914)	0.0715** (0.0327)
γ_4_(Treat)* (CP)	0.1568* (0.0873)	0.0721* (0.0393)	0.1214 (0.0988)	0.0954 (0.0832)	0.1223** (0.0571)	0.0735** (0.0351)
No. observations	953	953	953	665	665	665
Log-Likelihood	-917.35	-886.06	-1,086.04	-711.92	-659.26	-609.02
Weak Instrument F-Statistic Test	12.69 [0.0004]	13.81 [0.0003]	13.93 [0.0003]	11.75 [0.0006]	12.33 [0.0004]	12.16 [0.0004]
Wu-Hausman Endogeneity Test	-0.2385 (0.4081)	-0.1121 (0.1959)	-0.2365 (0.4951)	-0.0195 (0.1651)	-0.1451 (0.1057)	-0.7416 (0.5183)

Robust standard errors within parentheses, P-values within brackets,

***, ** and * indicate significance at 1%, 5% and 10% level. DV denotes dependent variable and CP stands for cultural participation.

In Table 10 we report the SURE estimates where the variable *treat* is taking a value of 1 whether the respondent has received the EU Blue card and 0 otherwise. We control in our regressions for various covariates mentioned in the methodology section, such as gender, age, education level, marital status, household income, household size and dummy variables for NUTS 1 area level. The results confirm the sharp and fuzzy RDD estimates in Tables 4–9, regarding the signs of the coefficients, indicating that the EU Blue Card holders are more likely to participate in the cultural activities we explore and more likely to report higher levels of SWB compared to those who are not holders. However, the estimates are mostly biased downwards, showing that the impact of the EU Blue Card programme is lower compared to the effects found in Tables 4–9.

**Table 10:** Ordered Probit SURE estimates.

**First Regression: Cultural Participation**	DV: Participation in Cinema and Jazz/Pop Concerts	DV: Participation in Classical Musical Performance, Opera or Theatre	DV: Practising Artistic Activities
β_0_ (Treat)	0.1510*** (0.0302)	0.3164*** (0.0184)	0.2376*** (0.0177)
**Second Regression: SWB**	DV: Life Satisfaction	DV: Life Satisfaction	DV: Life Satisfaction
γ_0_ (Treat)	0.4215*** (0.0511)	0.1442* (0.0762)	0.5119** (0.2448)
γ_3_(CP)	0.0479*** (0.0021)	0.1756*** (0.0107)	0.0435*** (0.0066)
γ_4_(Treat)* (CP)	0.0980** (0.0152)	0.0626* (0.0332)	0.0271 (0.0304)
No. observations	22,359	22,359	22,359
Log-Likelihood	-77,982.536	-63,033.212	-76,966.54

Robust standard errors within parentheses,

***, ** and * indicate significance at 1%, 5% and 10% level. DV denotes dependent variable and CP stands for cultural participation.

**Fig 4:**
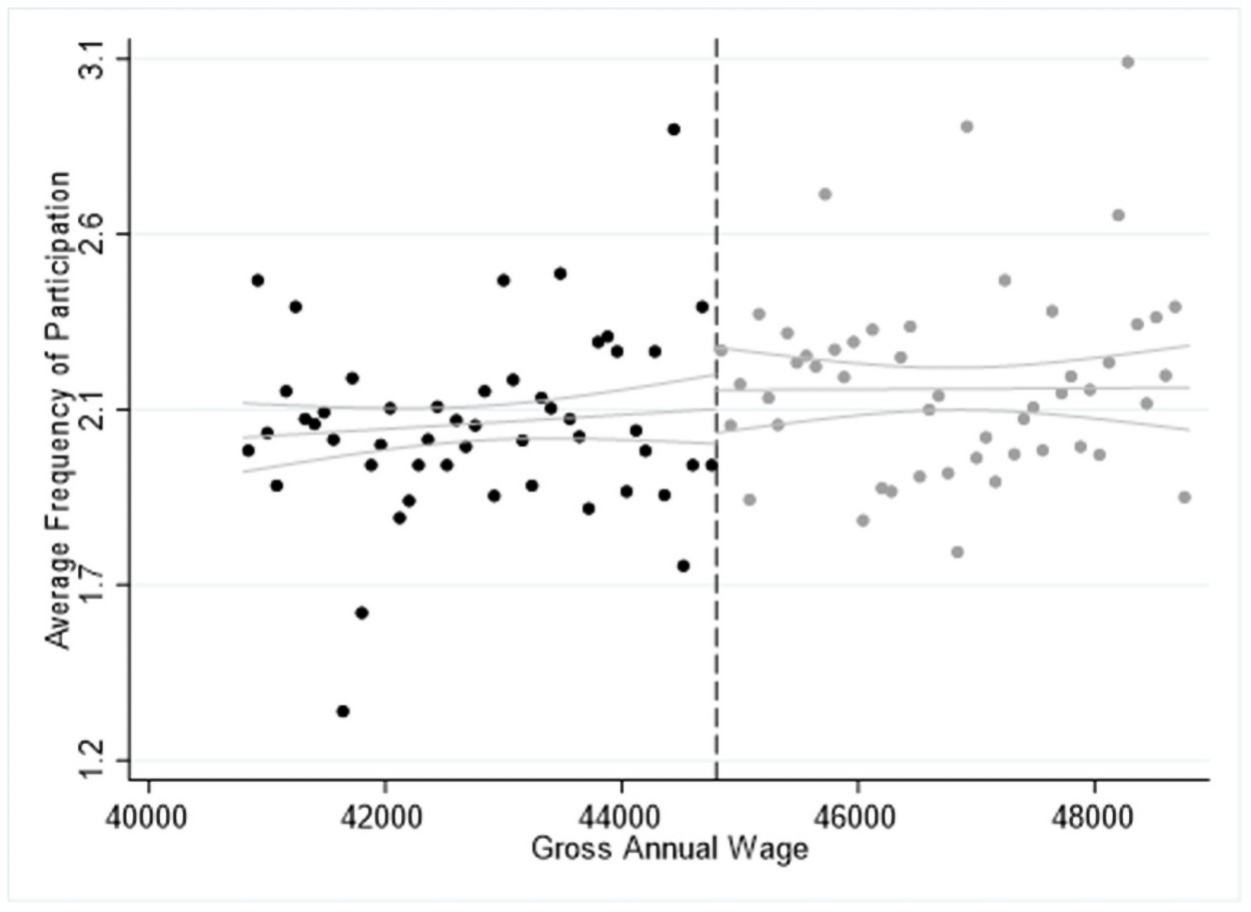
Sharp RDD estimates for participation in cinema and jazz/pop concerts using the salary threshold of 44,800€ and 2011 as the “Placebo” implementation year of the EU Blue Card programme.

**Fig 5:**
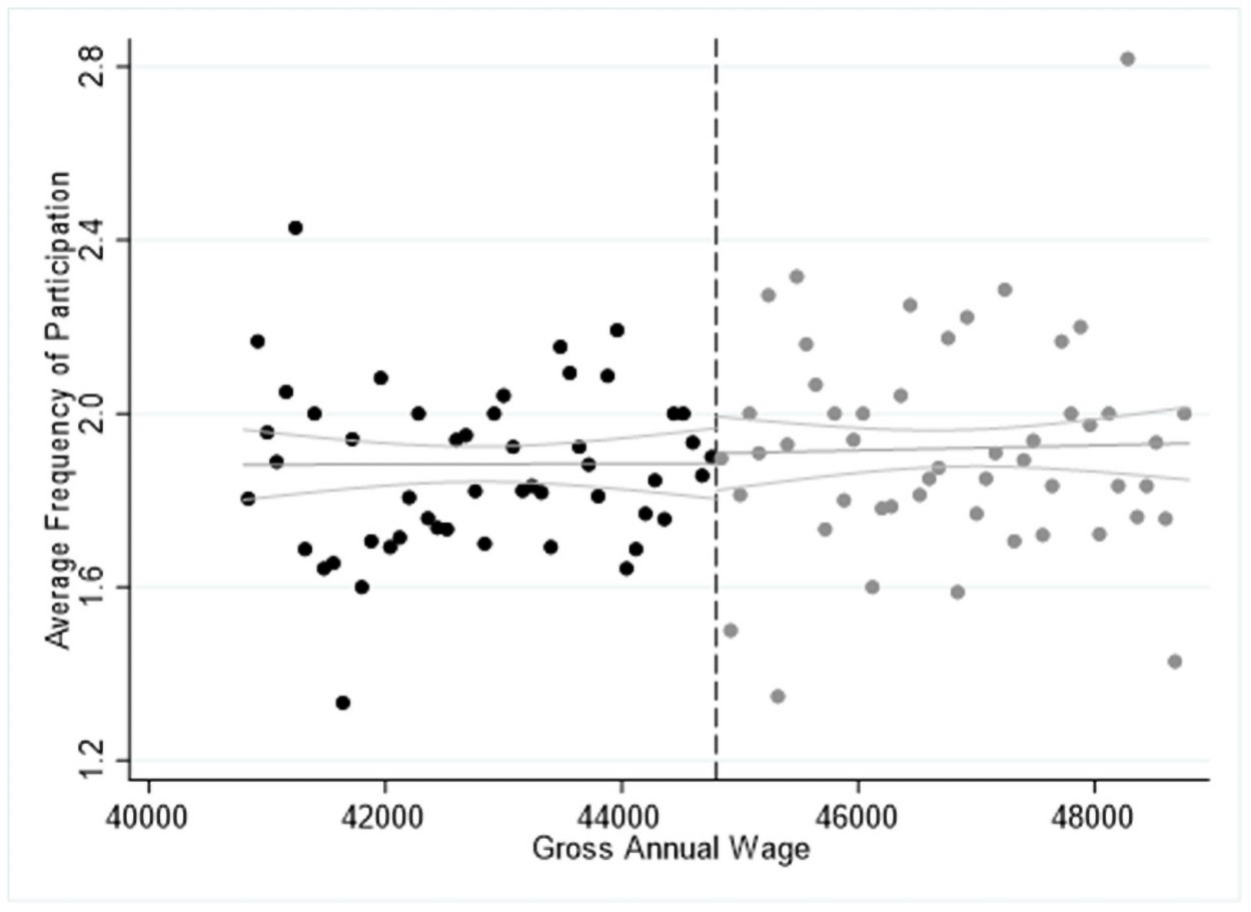
Sharp RDD estimates for participation in classical music performance, opera or theatre using the salary threshold of 44,800€ and 2011 as the “Placebo” implementation year of the EU Blue Card programme.

**Fig 6:**
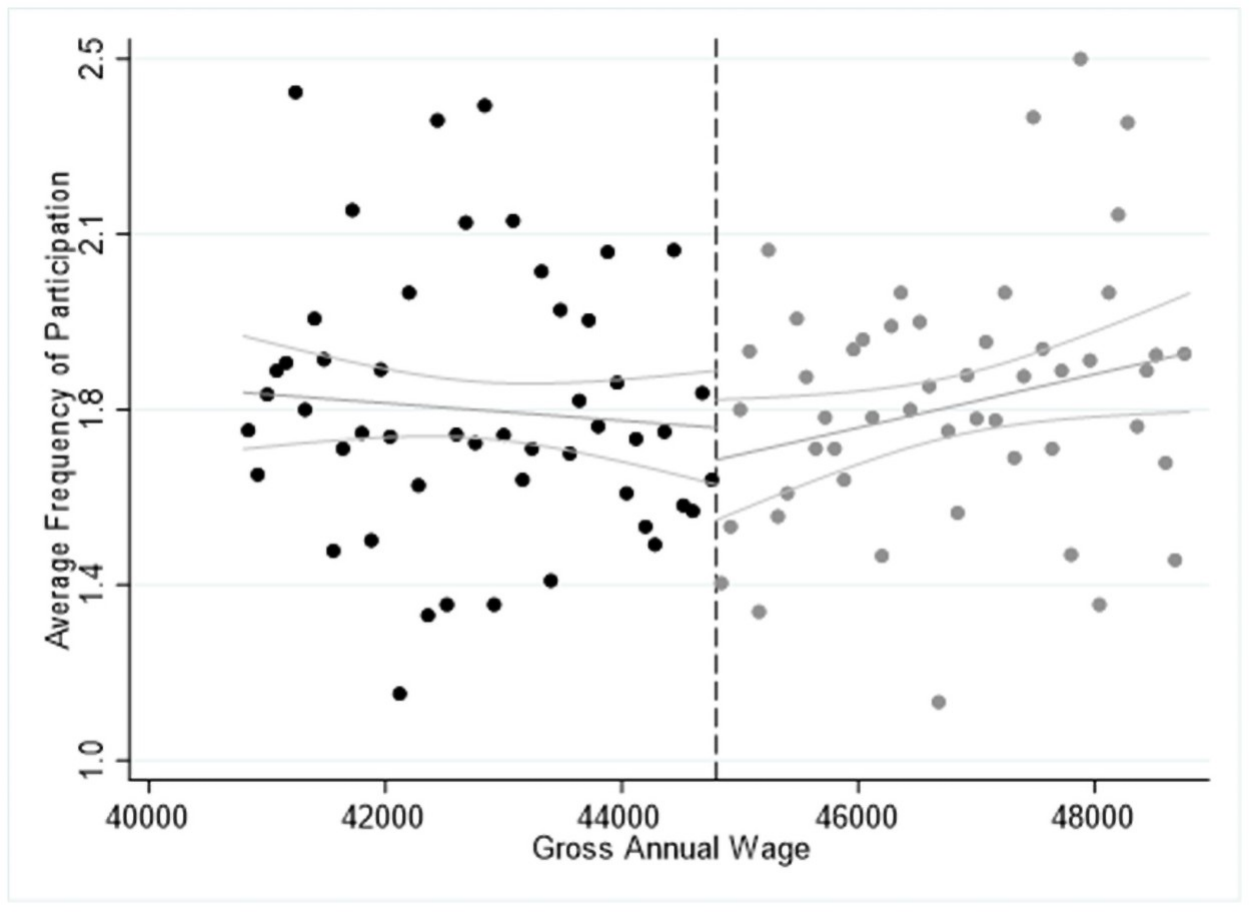
Sharp RDD estimates for participation in practising artistic activities using the salary threshold of 44,800€ and 2011 as the “Placebo” implementation year of the EU Blue Card programme.

In Table 11 we report the “placebo” sharp RDD estimates using the bandwidth of 4,000€ and the year of 2011 as the policy-reform year. In particular, we repeat the sharp RDD estimates considering only the year 2011, assuming that the EU Blue card programme was implemented in 2011 instead of 2012. In this case, the estimates of the main coefficients *β*_*0*_ for regression (1) and *γ*_*0*_ for regression (2) should become insignificant, or present the opposite sign of the estimates found in Tables 4–6, indicating that before the programme implementation, the non-EU/EEA first-generation immigrants were participating less frequently and were reporting lower levels of life satisfaction. In all cases we find the coefficients *β*_*0*_ and *γ*_*0*_ to be insignificant in both cultural participation and SWB regressions, indicating the robustness of our estimates in Tables 4–9. An exception is the negative coefficient *γ*_*0*_ in the participation in classical musical performance, opera or theatre for those employed in shortage occupations, and negative in all regressions for the high-skilled workers. This is also confirmed from Figs 4–6 where we illustrate the average participation in the cultural activities explored around the cut-off point of 44,800€ and bandwidth of 4,000€. In all three cases, we find no significant jump from the right side of the cut-off point, indicating that the average cultural participation of the first generation non-EU/EEA migrants was not significantly different from the average frequency of natives’ and first-generation EU migrants’ participation in 2011. The concluding remarks remain the same when we consider the bandwidths of 2,000€ and 3,000€ or when we consider the cut-off point or salary 34,944€, thus, we do not report the related figures. However, we should treat the results in Table 11 with caution, since we use the threshold values of 2012, while these values were not in force in 2011, as the EU Blue Card agreement was absent.

**Table 11:** Robust check: Sharp RDD estimates using as policy reform the year 2011, with uniform Kernel and bandwidth 4,000€.

**Panel A: Employed in Shortage Occupations**			
**First Regression: Cultural Participation**	DV: Participation in Cinema and Jazz/Pop Concerts	DV: Participation in Classical Musical Performance, Opera or Theatre	DV: Practising Artistic Activities
β_0_ (Treat)	-0.3704 (0.4768)	-0.2939 (0.2236)	-0.4961 (0.4766)
β_1_(wage -c)	-0.0018 (0.0041)	0.00016 (0.00046)	0.00032 (0.00048)
β_2_(Treat)* (wage -c)	-0.0025 (0.0021)	-0.00021 (0.00019)	-0.0018 (0.0019)
**Second Regression: SWB**	DV: Life Satisfaction	DV: Life Satisfaction	DV: Life Satisfaction
γ_0_ (Treat)	-3.9044 (2.6676)	-2.5372** (1.1473)	-0.8932 (0.7892)
γ_3_(CP)	0.3583*** (0.0821)	0.2032** (0.0862)	0.1317** (0.0621)
γ_4_(Treat)* (CP)	-0.8932 (0.7781)	-0.8779 (0.7582)	-0.2186 (0.2403)
No. observations	403	403	403
Log-Likelihood	-1,131.404	-1,081.680	-1,162.519
**Panel B: High Skilled**			
**First Regression: Cultural Participation**	DV: Participation in Cinema and Jazz/Pop Concerts	DV: Participation in Classical Musical Performance, Opera or Theatre	DV: Practising Artistic Activities
β_0_ (Treat)	-0.0759 (0.5701)	0.1429 (0.5257)	-0.1638 (0.5234)
β_1_(wage -c)	0.0054 (0.0042)	0.0048 (0.0068)	-0.0011 (0.0009)
β_2_(Treat)* (wage -c)	-0.0059 (0.0045)	-0.0051 (0.0047)	-0.0004 (0.0003)
**Second Regression: SWB**	DV: Life Satisfaction	DV: Life Satisfaction	DV: Life Satisfaction
γ_0_ (Treat)	-2.6166* (1.4151)	-2.6736* (1.3565)	-2.7216** (1.3132)
γ_3_(CP)	0.0357 (0.0874)	0.2525** (0.1205)	0.2197** (0.1041)
γ_4_(Treat)* (CP)	-0.4463 (0.3545)	-0.4385 (0.3719)	-0.4025 (0.3727)
No. observations	244	244	244
Log-Likelihood	-605.778	-608.064	-668.346

Robust standard errors within parentheses,

***, ** and * indicate significance at 1%, 5% and 10% level. DV denotes dependent variable.

### Robustness checks

In Table 12, we report the fuzzy RDD estimates across various demographic groups as robustness checks, and in particular, we present the estimates by native and migrant groups, gender and age. In the first panel, we report the estimates for those who are employed in the shortage occupations described in the methodology section. More specifically, in the first case, the treated group consists of the non-EU migrants who are granted the EU Blue Card, while in the second case the treated group remains the same, but the control group consists of the EU migrants. Based on the first regression estimates, we confirm hypothesis H_1_, where the EU Blue Card programme increases the participation of the treated subjects in the three cultural activities explored. The impact is higher in the participation in cinema and jazz-pop concerts, followed by practising artistic activities. Therefore, we observe that even though we have shown in Table 2 that non-EU migrants participate less frequently in all three cultural activities, the EU Blue Card programme increases the participation for both types of workers.

**Table 12:** Ordered Probit SURE FRDD robustness checks.

	**Employed in Shortage Occupations**: Treated Group Non-EU Migrants Blue Card Holders and Control Group German Natives			**Employed in Shortage Occupations**: Treated Group Non-EU Migrants Blue Card Holders and Control Group EU Migrants		
**First Regression: Cultural Participation**	Cinema and Jazz/Pop Concerts	Classical Musical Performance, Opera or Theatre	Practising Artistic Activities	Cinema and Jazz/Pop Concerts	Classical Musical Performance, Opera or Theatre	Practising Artistic Activities
β_0_ (Treat)	0.4267*** (0.0870)	0.1455** (0.0722)	0.2647*** (0.0898)	0.3921*** (0.0868)	0.1773** (0.0895)	0.3253*** (0.0915)
**Second Regression: SWB**	LS	LS	LS	LS	LS	LS
γ_0_ (Treat)	0.2751* (0.1406)	0.0984 (0.2292)	0.7947*** (0.2416)	0.2545** (0.1169)	0.7624** (0.3224)	0.3773 (0.2457)
γ_3_(CP)	0.2477*** (0.0366)	0.3163** (0.0384)	0.0396** (0.0188)	0.1104*** (0.0221)	0.2430*** (0.0278)	0.0448** (0.0191)
γ_4_(Treat)* (CP)	0.2114*** (0.0773)	0.1330* (0.0737)	0.0731* (0.0409)	0.0537 (0.0474)	0.0817** (0.0376)	0.0659 (0.0532)
No. observations	20,337	20,337	20,337	2,022	2,022	2,022
Log-Likelihood	-11,107.58	-10,967.31	-11,333.60	-18,481.15	-16,716.59	-18,407.06
**First Regression: Cultural Participation**	**High Skilled**: Treated Group Non-EU Migrants Blue Card Holders and Control Group German Natives			**High Skilled**: Treated Group Non-EU Migrants Blue Card Holders and Control Group EU Migrants		
β_0_ (Treat)	0.4240*** (0.0975)	0.4085*** (0.0991)	0.4156*** (0.1004)	0.2074 (0.1844)	0.2799* (0.1445)	0.4275** (0.1898)
**Second Regression: SWB**	LS	LS	LS	LS	LS	LS
γ_0_ (Treat)	0.0876 (0.2732)	0.4807* (0.2756)	0.5442** (0.2601)	0.4746 (0.4298)	0.4460* (0.2337)	0.9078** (0.4192)
γ_3_(CP)	0.1600*** (0.0387)	0.2841*** (0.0415)	0.1867*** (0.0256)	0.1652** (0.0778)	0.2532** (0.1131)	0.1599** (0.0759)
γ_4_(Treat)* (CP)	0.1046 (0.0863)	0.2732** (0.1369)	0.0591 (0.0586)	0.2115 (0.1359)	0.2168** (0.0970)	0.2538*** (0.0928)
No. observations	20,337	20,337	20,337	2,022	2,022	2,022
Log-Likelihood	-9,865.25	-9,737.39	-10,093.21	-2,151.88	-2,144.95	-2209.49
	**Employed in Shortage Occupations**: Males			**Employed in Shortage Occupations**: Females		
**First Regression: Cultural Participation**	Cinema and Jazz/Pop Concerts	Classical Musical Performance, Opera or Theatre	Practising Artistic Activities	Cinema and Jazz/Pop Concerts	Classical Musical Performance, Opera or Theatre	Practising Artistic Activities
β_0_ (Treat)	0.3672*** (0.1383)	0.0538 (0.1406)	0.0742 (0.1441)	0.4796*** (0.1107)	0.2005* (0.1120)	0.4350*** (0.1141)
**Second Regression: SWB**	LS	LS	LS	LS	LS	LS
γ_0_ (Treat)	0.1361 (0.3650)	0.1878 (0.4024)	0.9692*** (0.3624)	0.0452 (0.3178)	0.3539 (0.3595)	0.6410** (0.3134)
γ_3_(CP)	0.2536*** (0.0555)	0.3089*** (0.0572)	0.0244* (0.0127)	0.2186*** (0.0463)	0.3060*** (0.0489)	0.0497** (0.0225)
γ_4_(Treat)* (CP)	0.2210* (0.1195)	0.2081* (0.1263)	0.1285 (0.0810)	0.1762* (0.1008)	0.0861 (0.1112)	0.1032 (0.0888)
No. observations	10,968	10,968	10,968	11,391	11,391	11,391
Log-Likelihood	-9,728.481	-9,681.10	-9,795.85	-11,340.40	-11,249.10	-11,501.86
	**High Skilled**: Males			**High Skilled**: Females		
**First Regression: Cultural Participation**	Cinema and Jazz/Pop Concerts	Classical Musical Performance, Opera or Theatre	Practising Artistic Activities	Cinema and Jazz/Pop Concerts	Classical Musical Performance, Opera or Theatre	Practising Artistic Activities
β_0_ (Treat)	0.3535** (0.1447)	0.4373*** (0.1475)	0.3382** (0.1510)	0.4028*** (0.1265)	0.2901** (0.1278)	0.4724*** (0.1295)
**Second Regression: SWB**	LS	LS	LS	LS	LS	LS
γ_0_ (Treat)	0.4870 (0.4132)	0.4277 (0.4113)	0.9553** (0.3847)	0.3831 (0.3603)	0.4002* (0.2257)	0.1778 (0.3484)
γ_3_(CP)	0.1682** (0.0720)	0.1874*** (0.0537)	0.1189** (0.0544)	0.1865*** (0.0514)	0.3288*** (0.0555)	0.1557** (0.0645)
γ_4_(Treat)* (CP)	0.1201 (01329)	0.1129 (0.1294)	0.1309 (0.0863)	0.1724* (0.1002)	0.1976* (0.1154)	0.0593 (0.0788)
No. observations	10,968	10,968	10,968	11,391	11,391	11,391
Log-Likelihood	-9,143.9318	-9,085.265	-10,093.21	-9,253.0247	-9,383.696	-10,200.286
	**Shortage Occupations**: 16–44 Age			**Employed in Shortage Occupations**: 45+ Age		
**First Regression: Cultural Participation**	Cinema and Jazz/Pop Concerts	Classical Musical Performance, Opera or Theatre	Practising Artistic Activities	Cinema and Jazz/Pop Concerts	Classical Musical Performance, Opera or Theatre	Practising Artistic Activities
β_0_ (Treat)	0.3703*** (0.1178)	0.0178 (0.1200)	0.3028** (0.1401)	0.4665*** (0.1405)	0.2704* (0.1399)	0.2199 (0.1456)
**Second Regression: SWB**	LS	LS	LS	LS	LS	LS
γ_0_ (Treat)	0.3278** (0.1580)	0.2258 (0.1529)	0.9348*** (0.3219)	0.1210 (0.1581)	0.2521 (0.2806)	0.6482* (0.3542)
γ_3_(CP)	0.1616*** (0.0515)	0.2263** (0.0659)	0.0353** (0.0162)	0.2931*** (0.0541)	0.3623*** (0.0530)	0.0422** (0.0201)
γ_4_(Treat)* (CP)	0.2721*** (0.1032)	0.1114** (0.0556)	0.1302* (0.0721)	0.1133* (0.0628)	0.0707 (0.1478)	0.0547 (0.0886)
No. observations	11,627	11,627	11,627	10,732	10,732	10,732
Log-Likelihood	-9,608.00	-9,527.82	-9,741.81	-6,297.787	-9,306.29	-9,432.03
	**High Skilled**: 16–44 Age			**High Skilled**: 45+ Age		
**First Regression: Cultural Participation**	Cinema and Jazz/Pop Concerts	Classical Musical Performance, Opera or Theatre	Practising Artistic Activities	Cinema and Jazz/Pop Concerts	Classical Musical Performance, Opera or Theatre	Practising Artistic Activities
β_0_ (Treat)	0.2490* (0.1349)	0.1891 (0.1366)	0.3604*** (0.1367)	0.3935*** (0.1785)	0.4887*** (0.1494)	0.2746* (0.1520)
**Second Regression: SWB**	LS	LS	LS	LS	LS	LS
γ_0_ (Treat)	0.3788** (0.1744)	0.2201* (0.1255)	0.4111 (0.3619)	0.1734 (0.4665)	0.9518** (0.4426)	0.7066* (0.4044)
γ_3_(CP)	0.1341* (0.0719)	0.1622** (0.0795)	0.0333* (0.0187)	0.1462** (0.0601)	0.3026*** (0.0613)	0.0526** (0.0257)
γ_4_(Treat)* (CP)	0.2068* (0.1231)	0.1044 (0.1251)	0.0500 (0.0825)	0.1160 (0.1414)	0.1207** (0.0546)	0.1226 (0.0908)
No. observations	11,627	11,627	11,627	10,732	10,732	10,732
Log-Likelihood	-8,554.34	-8,506.50	-8,696.01	-8,644.13	-8,619.71	-10,200.286

Robust standard errors within parentheses,

***, ** and * indicate significance at 1%, 5% and 10% level. LS denotes life satisfaction.

In all cases, we confirm hypothesis H_2_, where participation in those cultural activities improves the life satisfaction of both natives and migrants. When we consider the EU Blue Card holders and German natives, participation in the EU Blue Card programme improves the life satisfaction of the treated subjects more than the SWB of natives, confirming hypothesis H_3_, with the largest impact in the regression of participation in cinema and jazz-pop concerts, followed by participation in theatrical play or opera, and practising activities. On the other hand, when we consider EU and non-EU migrants, the estimates confirm hypothesis H_3_ only in the case of participation in theatre or opera. Previous studies have addressed the importance of socio-cultural participation in the SWB of migrants. For instance, old aged first-generation migrants in the Czech Republic participate less frequently in various socio-cultural activities, such as attendance to the theatre, classical music performance, concerts, sports events and visits to museums. However, those who participate experience higher changes in their SWB compared to the natives [46]. Creative and cultural activities were found to have positive effects on the psychosocial well-being of refugees and asylum seekers [52].

Regarding the estimates by gender we observe in both types of workers, females are more likely to participate in the cultural activities explored, while in the case of males employed in shortage occupations, the EU Blue Card programme has no impact on their participation in theatre, classical music performance or opera, and practising artistic activities. We should highlight that in this case the treated and control subjects are defined as in the estimates of Tables 4–9. To recall, treated subjects are the non-EU migrants participating in the EU Blue card programme, while the control subjects are German natives and EU migrants. Regarding the impact on life satisfaction, we find that the EU Blue Card programme has the largest impact for females practising in cinema, theatre and opera, while for males practising artistic activities improves their life satisfaction. Nevertheless, when we test hypothesis H_3_ women participating in jazz-pop concerts, cinema and theatre, report higher positive changes in their life satisfaction compared to the control subjects, while no difference is found in the life satisfaction between treated and control subjects when we consider the male sample.

This is consistent with earlier studies that found women are more likely to participate in “highbrow” activities, such as visits to museums, concerts and theatrical plays [53, 54]. In comparison to male participants, women who attend arts events are more likely to be inspired by the artistic value of a performance and a desire to learn more [55]. Furthermore, women from an early age, rely on artistic lessons to create networks of friends, and social network characteristics, especially network diversity, are better predictors of women’s cultural participation than of men’s [56]. However, this may depend on the education level, as more educated men tend to participate more in “highbrow” cultural activities, while women present higher cultural consumption in younger age groups [57]. This is confirmed by the high-skilled workers, where we find a positive impact of the EU Blue Card programme on both males and females, with men preferring the attendance to theatre and opera, and women participating more in cinema, concerts and practising artistic activities. Furthermore, the German society can be considered as a feminine one, compared with the origin countries of some non-EU migrants. Thus, non-EU female migrants coming to Germany are more likely to experience higher changes in their SWB, especially the high-skilled. Moreover, feminine societies may offer to both male and female migrants, and especially female, more opportunities for the fulfilment of multiple social roles, promoting social inclusion and encouraging participation in cultural activities that are associated with network satisfaction and higher levels of SWB [58, 59].

We should notice that according to the literature, “lowbrow” activities refer to activities related to low taste and low intellectual interest, such as reading popular magazines, and attendance at sports events and religious activities. On the other hand, “highbrow” activities are related to and are defined as the selections among the available alternatives that have the air of being the noblest and rarest and which provide the opportunity to gain most distinction on the prevailing cultural markets, favoured by those who have attained the highest education level and belong to the high-income groups [30, 31, 60].

### Summary of the results

In Tables 4–7 we have reported the estimated of the benchmark model; the Ordered Probit SURE within a sharp RDD framework, while in Table 10 we presented the estimates of the Ordered Probit SURE. In Table 13 we report the aggregated results of our favoured estimates derived from the fuzzy RDD using the full sample in Table 8, and the estimates across native and migrants, gender and age groups, reported in Table 12. When we consider the total sample, we find a positive impact of the EU Blue Card Programme on the participation of the treated subjects for both types of workers; the high-skilled and those employed in the shortage occupations, indicated by Treat (EU Blue Card). In particular, the programme increases the participation of the non-migrants more than the control group, which is German natives, EU migrants and non-EU migrants that are not eligible for the programme. Then we report the direction and significance between life satisfaction and participation in each cultural activity. In this case, we find a positive impact of the EU Blue Card Programme on the SWB of those employed in shortage occupations, through participation in cinema, jazz-pop concerts, theatre or opera, but no impact is found when they practise artistic activities.

**Table 13:** Summary of the FRDD results.

**Panel A: Total Sample**				
**Employed in Shortage Occupations**	Cinema and Jazz/Pop Concerts	Classical Musical Performance, Opera or Theatre	Practising Artistic Activities	Life Satisfaction
Treat (EU Blue Card)	ꜛ	ꜛ	ꜛ	
Treat and Participation in Cinema, Jazz/Pop Concerts				ꜛ
Treat and Participation in Opera or Theatre				ꜛ
Treat and Practising Artistic Activities				**Insignificant**
**High Skilled**	Cinema and Jazz/Pop Concerts	Classical Musical Performance, Opera or Theatre	Practising Artistic Activities	Life Satisfaction
Treat (EU Blue Card)	ꜛ	ꜛ	ꜛ	
Treat and Participation in Cinema, Jazz/Pop Concerts				**Insignificant**
Treat and Participation in Opera or Theatre				ꜛ
Treat and Practising Artistic Activities				ꜛ
**Panel B: Non-EU Migrants and German Natives**				
**Employed in Shortage Occupations**	Cinema and Jazz/Pop Concerts	Classical Musical Performance, Opera or Theatre	Practising Artistic Activities	Life Satisfaction
Treat (EU Blue Card)	ꜛ	ꜛ	ꜛ	
Treat and Participation in Cinema, Jazz/Pop Concerts				ꜛ
Treat and Participation in Opera or Theatre				ꜛ
Treat and Practising Artistic Activities				ꜛ
**High Skilled**	Cinema and Jazz/Pop Concerts	Classical Musical Performance, Opera or Theatre	Practising Artistic Activities	Life Satisfaction
Treat (EU Blue Card)	ꜛ	ꜛ	ꜛ	
Treat and Participation in Cinema, Jazz/Pop Concerts				**Insignificant**
Treat and Participation in Opera or Theatre				ꜛ
Treat and Practising Artistic Activities				**Insignificant**
**Panel C: Non-EU Migrants and EU Migrants**				
**Employed in Shortage Occupations**	Cinema and Jazz/Pop Concerts	Classical Musical Performance, Opera or Theatre	Practising Artistic Activities	Life Satisfaction
Treat (EU Blue Card)	ꜛ	ꜛ	ꜛ	
Treat and Participation in Cinema, Jazz/Pop Concerts				**Insignificant**
Treat and Participation in Opera or Theatre				ꜛ
Treat and Practising Artistic Activities				**Insignificant**
**High Skilled**	Cinema and Jazz/Pop Concerts	Classical Musical Performance, Opera or Theatre	Practising Artistic Activities	Life Satisfaction
Treat (EU Blue Card)	**Insignificant**	ꜛ	ꜛ	
Treat and Participation in Cinema, Jazz/Pop Concerts				**Insignificant**
Treat and Participation in Opera or Theatre				ꜛ
Treat and Practising Artistic Activities				ꜛ
**Panel D: Males**				
**Employed in Shortage Occupations**	Cinema and Jazz/Pop Concerts	Classical Musical Performance, Opera or Theatre	Practising Artistic Activities	Life Satisfaction
Treat (EU Blue Card)	ꜛ	**Insignificant**	**Insignificant**	
Treat and Participation in Cinema, Jazz/Pop Concerts				ꜛ
Treat and Participation in Opera or Theatre				ꜛ
Treat and Practising Artistic Activities				**Insignificant**
**High Skilled**	Cinema and Jazz/Pop Concerts	Classical Musical Performance, Opera or Theatre	Practising Artistic Activities	Life Satisfaction
Treat (EU Blue Card)	ꜛ	ꜛ	ꜛ	
Treat and Participation in Cinema, Jazz/Pop Concerts				**Insignificant**
Treat and Participation in Opera or Theatre				**Insignificant**
Treat and Practising Artistic Activities				**Insignificant**
**Panel E: Females**				
**Employed in Shortage Occupations**	Cinema and Jazz/Pop Concerts	Classical Musical Performance, Opera or Theatre	Practising Artistic Activities	Life Satisfaction
Treat (EU Blue Card)	ꜛ	ꜛ	ꜛ	
Treat and Participation in Cinema, Jazz/Pop Concerts				ꜛ
Treat and Participation in Opera or Theatre				**Insignificant**
Treat and Practising Artistic Activities				**Insignificant**
**High Skilled**	Cinema and Jazz/Pop Concerts	Classical Musical Performance, Opera or Theatre	Practising Artistic Activities	Life Satisfaction
Treat (EU Blue Card)	**Insignificant**	ꜛ	**Insignificant**	
Treat and Participation in Cinema, Jazz/Pop Concerts				ꜛ
Treat and Participation in Opera or Theatre				ꜛ
Treat and Practising Artistic Activities				**Insignificant**
**Panel F: 16–44 Years Old**				
**Employed in Shortage Occupations**	Cinema and Jazz/Pop Concerts	Classical Musical Performance, Opera or Theatre	Practising Artistic Activities	Life Satisfaction
Treat (EU Blue Card)	ꜛ	**Insignificant**	ꜛ	
Treat and Participation in Cinema, Jazz/Pop Concerts				ꜛ
Treat and Participation in Opera or Theatre				ꜛ
Treat and Practising Artistic Activities				ꜛ
**High Skilled**	Cinema and Jazz/Pop Concerts	Classical Musical Performance, Opera or Theatre	Practising Artistic Activities	Life Satisfaction
Treat (EU Blue Card)	ꜛ	**Insignificant**	ꜛ	
Treat and Participation in Cinema, Jazz/Pop Concerts				ꜛ
Treat and Participation in Opera or Theatre				**Insignificant**
Treat and Practising Artistic Activities				**Insignificant**
**Panel G: 45+ Years Old**				
**Employed in Shortage Occupations**	Cinema and Jazz/Pop Concerts	Classical Musical Performance, Opera or Theatre	Practising Artistic Activities	Life Satisfaction
Treat (EU Blue Card)	ꜛ	ꜛ	**Insignificant**	
Treat and Participation in Cinema, Jazz/Pop Concerts				ꜛ
Treat and Participation in Opera or Theatre				**Insignificant**
Treat and Practising Artistic Activities				**Insignificant**
**High Skilled**	Cinema and Jazz/Pop Concerts	Classical Musical Performance, Opera or Theatre	Practising Artistic Activities	Life Satisfaction
Treat (EU Blue Card)	ꜛ	ꜛ	ꜛ	
Treat and Participation in Cinema, Jazz/Pop Concerts				**Insignificant**
Treat and Participation in Opera or Theatre				ꜛ
Treat and Practising Artistic Activities				**Insignificant**

Therefore, this implies that the treated subjects who participate for instance, in cinema- indicated by the component *Treat and Participation in Cinema*, *Jazz/Pop Concerts* in the Panel A of Table 12- experience higher positive changes in their life satisfaction compared to the control group, but no differences in the SWB are noted when they practise artistic activities. Similarly, the high-skilled experience higher changes in their SWB when they participate in theatre or opera and artistic activities, but the differences become statistically insignificant when they participate in cinema and jazz-pop concerts. As we have discussed earlier, these differences can be explained by the theory of cultural capital developed by Bourdieu [30, 31], arguing that those belonging in high-income groups, in higher social class and are more educated are more likely to participate in passive “highbrow” activities, such as attendance to theatre and opera, and are also more likely to engage in active participation, such as practising artistic activities.

In panels B and C, we summarise the findings when the control groups are respectively the German natives and the EU-migrants, while the treated group comprises the non-EU migrants participating in the EU-Blue Card programme. In all cases, we find a positive impact of the programme on the participation of the treated subjects, while they experience higher positive changes in their life satisfaction in all cases when they are employed in the shortage occupations and the control group are German natives. High-skilled workers do not experience significantly different levels of life satisfaction compared to the control group, except for the participation in theatre or opera. The same applies to those employed in shortage occupations, but the control group comprises EU migrants. To recall the hypothesis H_3_ we should highlight that these findings do not imply that cultural participation is not positively related to life satisfaction, but they show that the changes in the SWB of the treated subjects due to cultural participation caused from the EU-Blue Card programme are not statistically different from the changes experienced by the respondents in the control group.

In panels D-E we report the impact of the EU Blue Card on male and female participation, which is significant only in the case of cinema and jazz-pop concerts, while it has a positive impact on women’s participation in all cultural activities explored. As we have discussed in the previous section, women tend to attend “highbrow” activities more often compared to their male counterparts. However, this impact is mediated when we consider the education [57], where males participate more often in both passive “lowbrow” and “highbrow” activities, and they also participate in active events such as practising artistic activities. Nevertheless, the results show that the EU programme had a positive impact on females’ life satisfaction through cultural participation, in both groups of workers, while it affects the SWB only of males employed in the shortage occupation. This could be potentially explained by the higher inspirations and expectations of high-skilled males, where participation in the EU Blue Card programme and in cultural activities does not significantly increase their SWB more compared to the male non-participants in the EU Blue Card programme.

In the last two panels, F and G, of Table 13, we report the estimates by the age groups. It is interesting that treated subjects belonging in the age group of 15–44 years’ old, are more likely to participate in cinema and jazz-pop concerts, and to practise artistic activities, but the EU Blue Card programme has no impact on their participation in theatre or opera related activities. However, those employed in shortage occupations experience higher positive changes in their life satisfaction, compared to the high-skilled workers who improve their life satisfaction only through participation in cinema and jazz-pop concerts. On the contrary, the age group of 45 and older are more likely to participate in the cultural activities explored, because of the EU Blue Card programme, but report no differences in the improvement of their SWB compared to the control group except for those employed in shortage occupations and participate in cinema, and jazz-pop concerts, and the high-skilled participating in opera or theatre. These results can be explained, as we have highlighted earlier, by the higher expectations of the high-skilled or the older respondents that could not be probably met [39, 40].

Overall, the results vary by the socio-economic and demographic group the respondents belong to, as we find that high-skilled participate more in “highbrow” activities and those employed in shortage occupation participate more in cinema and concerts because of the EU Blue Card programme. Thus, apart from the needs in the German economy, and whether positions in shortage occupations or high-skilled professions can be filled by the natives, EU or non-EU migrants, the results show that the EU Blue Card programme has overall a positive impact on the participation in cultural activities across various groups. However, those employed in shortage occupations express a higher change in their life satisfaction, implying that they can be also more productive according to previous studies arguing that well-being improves productivity [61]. The same concluding remarks also hold, when we decompose the analysis by gender, where both men and women employed in shortage occupations experience higher levels of life satisfaction, while high-skilled women are also more likely to report higher levels of SWB. Regarding age, we find the programme had a positive impact on the participation in both age groups explored; however, the younger- those aged between 16-44- employed in the shortage occupations report higher levels of SWB. Therefore, depending on the demand and labour supply for certain job positions and if the target is to improve productivity, the programme may focus mainly on the younger immigrants employed in shortage occupations, as they are more likely to experience higher levels of life satisfaction compared to their older and high-skilled counterparts. Since well-being is associated with higher levels of productivity and job-firm performance [61], the policymakers may explore the case of shortage occupations.

A limitation of this study lies in the structure of the GSOEP, where the information about whether the respondent has acquired the EU Blue card is available only in 2018. Moreover, no related information is recorded before 2012 that would allow us to establish a causal inference by implementing a standard Difference-in-Differences (DiD) framework. In particular, we could define as the treated group the non-EU migrants who have gained the EU Blue Card, considering various control groups, including German natives, EU migrants and non-EU migrants who are not eligible or have not acquired the EU Blue Card. Nevertheless, we suggest that related information on migration policies and residence permits for migrants should be recorded in future waves of the GSOEP survey. Furthermore, we should notice that in the sharp RDD estimates the take‐up rates or the rates of participation in the EU Blue Card programme could be well below 100 percent. This implies that not all individuals who are eligible ever participate or apply for it since there is no related information in the GSOEP as we mentioned before. Hence, our results regarding the sharp RDD should be interpreted as intention‐to‐treat (ITT) effects. Regarding the fuzzy RDD, as we have highlighted earlier about the DiD framework, we have limited our analysis only to one year.

## Discussion and conclusion

The findings of this research have implications for policymakers and researchers, as the integration of immigrants is facilitated by employment opportunities and income. Policymakers should therefore be mindful that growth and economic opportunities will draw migration and facilitate faster and more in-depth integration. In other words, the quality of life will inspire immigrants to remain in the country, to integrate into social, economic and cultural spheres, and to experience higher levels of well-being. According to the findings, the EU Blue Card programme has provided employment opportunities and high potential earnings for high-skilled non-EU/EEA migrants, offering courses in language and cultural orientation and, above all, a permanent residence permit. These, in turn, had a positive effect on the frequency of participation in cultural activities for migrants and subsequently increased the SWB.

However, in practice, the EU Blue Card is a kind of puzzle, since although the Member States confront similar challenges in terms of an ageing population and labour shortages, their response to a harmonized highly skilled and profoundly talented immigration policy arrangement shifts impressively. Their national self-interests and their hesitance to relinquish sovereignty on migration matters, coupled with their fear of an overpowering wave of low-skilled migrants flowing into Europe, driven to a largely insignificant European migration scheme failing to attract high-skilled migrants that represent the limit to encourage further integration. Thus, instead of representing a signal to profoundly talented migrants and a figure of competition for the EU, the Blue Card illustrates the challenges to more prominent integration and the hesitance of Member States to relinquish more responsibility to the EU level.

More investigation about the impact of the EU Blue Card on cultural participation and SWB in other member states is required. For instance, the conditions in Sweden are clear-cut, where the low number of Blue Cards is a result of the liberal national approach, which is a far more inviting option for the non-EU migrants compared to the criteria of the Blue Card scheme. In particular, the national framework does not impose any sort of minimum requirements nor impediments, whereas providing migrants with rights as favourable as the ones entitled by the Blue Card, such as the plausibility to be unemployed for three months without losing the residence right and the rights granted to family members. These criteria make the national scheme less complicated and much more appealing for non-EU nationals wishing to enter Sweden for work regardless of being high-skilled or not [62]. On the other hand, in Germany and Netherlands, the EU Blue Card scheme provides more advantages for the high-skilled migrants willing to move to the country for employment reasons, and the scheme provided more beneficial arrangements than the national Directive scheme [62].

Overall, the purpose of the Blue Card Order is to make the EU more appealing to non-EU highly qualified workers and to solidify its competitiveness within the worldwide setting as well as its economic growth, but it had failed to meet its ambitions. The main reason for the huge contrasts within the numbers of EU Blue Cards granted are the size, economic structure and conditions of the single Member States, as well as the attractiveness of the European labour market, especially after the recession following the global financial crisis of 2007–2008, but they cannot fully explain the wide varieties and the disappointing low numbers in some Member States.

The single policies choices adopted from the Member States when implementing the European Directive may moreover offer further explanation since each country promoted and applied the EU Blue Card scheme distinctively. As it was illustrated, the diverse responses to the Blue Card as well as the extraordinary variety within the number of Blue Cards allowed do not depend only on the labour migration approach pursued by each country. For instance, Germany and the Netherlands are two nations sharing very similar characteristics, as their common approach towards labour migration is considered very restrictive, while they provide generous admission rules in favour of high-skilled migrants. However, the number of Blue Cards issued in Germany is countless times greater than that of the Netherlands, implying that other factors determining the number of EU Blue Cards granted and which deserve more study. Overall, to achieve a higher degree of harmonization in the labour migration and to create a real common market in the EU for non-EU nationals, a more exclusive role of the Blue Card as an instrument at the costs of the single national schemes should be granted [62].

Furthermore, an interesting avenue for future research is to explore how the migrants’ country of origin characteristics, such as linguistic and cultural distance, can affect the tendency of migrants to engage and participate in various cultural activities, and how these influence their SWB. Moreover, it is important to note that the foregoing findings are primarily focused on Germany, a country with a long history of migration and thus, a longer history of policy-research dialogues. Hence, it would be important and interesting to pose the same questions for relatively new migrant countries, such as the Southern European countries that first witnessed large-scale immigration in the 1990s, and the Syrian civil war and the large influx of refugees over the past 10 years. Moreover, it would be interesting to investigate the Central and Eastern European countries which introduced the EU migration and integration policy regime only after they acceded to the EU in 2004 and 2007. Since most of these countries still have low immigration experience and lack historically embedded national integration models, we will be able to observe from the very beginning how research-policy relations and structures have evolved there. Apart from the participation in cultural activities explored in this study, future research should extend and analyze the role of participation in a larger set of socio-cultural activities, including attendance at sports events; being a member of a community or political party; doing sports and participating in voluntary work, among others.

Overall, policies that facilitate participation in cultural activities would allow immigrants to integrate and develop their SWB into the social norms of the host societies. Policies should include investments in social inclusion and integration, especially as integration is a multi-faceted and multigenerational process. Immigration would enrich recipient countries and integration processes that facilitate collaborative and mutual learning and have the ability to create communities that are more inclusive, safe and happier.

However, the COVID-19 pandemic was a culture shock, and it had a huge impact on cultural participation because venue-based sectors, such as performing arts, live concerts, theatre, cinema and museums were completely shut down or were the hardest hit by social distancing measures. The findings of this study suggest that participation in cultural activities is a great opportunity for enjoyment, learning, social engagement and self-actualization for migrants, among other groups. Intangible cultural participation and heritage may be a source of social and psychological resilience during times of crises, such as the coronavirus pandemic. In these conditions, peoples’ access to cultural activities is significantly limited with adverse effects on the SWB. This can be especially the case of the migrants, as the findings show that participation in cultural activities is associated with higher positive changes in the SWB of the EU Blue Card holders compared to the German natives or EU migrants.

Overall, access to cultural activities must be ensured because it will help people to cope with the psychological trauma caused by crises and ensure their well-being during the recovery process. Fostering diversity of cultural expressions is an important way to cope with post-crisis trauma and bring impacted groups together. The findings imply that shutting down access to cultural activities and venues during the lockdown periods because of the COVID-19 pandemic will have a significant negative impact on the SWB. Therefore, policymakers, taking lessons from the current pandemic, should be aware of the importance of intangible cultural heritage, the role of participation in cultural activities, and the importance of cultural and creative sectors in the SWB and fostering long-term, inclusive recovery with complete community ownership.

The COVID-19 pandemic has exposed profound disparities, especially in cities, causing certain areas and individuals to be unfairly impacted. Apart from the migrants explored in this study, other groups have been affected by the pandemic as well, including ethnic minorities, disabled, widowed, and other marginalized groups. Strengthening economic and social security for cultural organizations, and practitioners will go a long way toward ensuring cultural freedom and inclusion. Arts and culture are important vehicles for telling stories, engaging communities, and fostering collaborative experiences. This helps societies in developing a common capacity for healing, resilience, and social cohesion following a crisis.

## Supporting information

S1 File (DOCX)
