# Supplementary material for: Impact of the EU Blue Card programme on cultural participation and subjective well-being of migrants in Germany
Source: PLoS One. 2021 Jul 12;16(7):e0253952. doi: 10.1371/journal.pone.0253952 (PMC8274866; doi:10.1371/journal.pone.0253952)
Supplement: S1 File — (DOCX) [file pone.0253952.s001.docx]

**Replication File**

use "D:\..........\ge.dta", clear

** Remove Duplicates

duplicates drop pid syear, forc

xtset pid syear

set matsize 5000

*** Keep only aged 16 and above, since the questions about participation and life satisfaction are not available.

*** Moreover, the EU BLUE Card programme refers to those in the labour force, which excludes those younger than 16 years old.

** We define the EU and non-EU status, 1 for EU and 0 for non-EU

gen eu=.

replace eu=1 if corigin>1 & (corigin>3 & corigin<18)

replace eu=1 if corigin>1 & (corigin==21 | corigin==22 | corigin==26 | corigin==28 | corigin==29 | corigin==31)

replace eu=1 if corigin==71 | corigin==101 | corigin==103 | corigin==116 | corigin==117 | corigin==118 | corigin==119 | corigin==122 | corigin==123

replace eu=0 if eu==. & corigin>1

** We prepare the sample for the first type of workers, which is the shortage occupations. According to the programme the ISCO is between 2000 and 4000

gen sample=1 if pgisco88>2000 & pgisco88<4000

gen c_yincome2012low=labourincome-34944 if syear==2012 & sample==1

replace c_yincome2012low=labourincome-36192 if syear==2013 & sample==1

replace c_yincome2012low=labourincome-37128 if syear==2014 & sample==1

replace c_yincome2012low=labourincome-37753 if syear==2015 & sample==1

replace c_yincome2012low=labourincome-38688 if syear==2016 & sample==1

replace c_yincome2012low=labourincome-39624 if syear==2017 & sample==1

replace c_yincome2012low=labourincome-40560 if syear==2018 & sample==1

** Then for the high-skilled and specifically those with a university degree

gen c_yincome2012=labourincome-44800 if syear==2012 & pgisced97==6

replace c_yincome2012=labourincome-46400 if syear==2013 & pgisced97==6

replace c_yincome2012=labourincome-47600 if syear==2014 & pgisced97==6

replace c_yincome2012=labourincome-48400 if syear==2015 & pgisced97==6

replace c_yincome2012=labourincome-49600 if syear==2016 & pgisced97==6

replace c_yincome2012=labourincome-50800 if syear==2017 & pgisced97==6

replace c_yincome2012=labourincome-52000 if syear==2018 & pgisced97==6

** Then create the treated thich is the non-EU migrants above the threshold, and control those with an income lower than the cutoff point

gen treatlow=1 if c_yincome2012low>=0 & eu==0

replace treatlow=0 if c_yincome2012low<0

*** Similarly for the high-skilled

gen treat=1 if c_yincome2012>=0 & eu==0

replace treat=0 if c_yincome2012<0

** Take the interaction between treat and the centered forcing variable (income) for both types of workers

gen treat_z2012low=treatlow*c_yincome2012low

gen treat_z2012=treat*c_yincome2012

** Take the quadratic term to check the quadratic specification of the RDD for both types of workers

gen c_yincome2012low2=c_yincome2012low*c_yincome2012low

gen c_yincome20122=c_yincome2012*c_yincome2012

** Take the interaction between treat and the quadratic centered forcing variable (income)

gen treat_z20122low=treatlow*c_yincome2012low2

gen treat_z20122=treat*c_yincome20122

*** Then we run the sharp RDD regressions in table 4 without covariates. You may include them

cmp setup

**Shortage occupations and bandwidth 2,000 Euros

xi:cmp (cinema_con= treatlow c_yincome2012low treat_z2012low ) ///

(life_sat= c.treatlow##c.cinema_con c_yincome2012low treat_z2012low ) if (c_yincome2012low >= -2000 & c_yincome2012low <= 2000) & syear>2011 , ind( $cmp_oprobit $cmp_oprobit) qui robust

**Shortage occupations and bandwidth 3,000 Euros

xi:cmp (cinema_con= treatlow c_yincome2012low treat_z2012low ) ///

(life_sat= c.treatlow##c.cinema_con c_yincome2012low treat_z2012low ) if (c_yincome2012low >= -3000 & c_yincome2012low <= 3000) & syear>2011 , ind( $cmp_oprobit $cmp_oprobit) qui robust

**Shortage occupations and bandwidth 4,000 Euros

xi:cmp (cinema_con= treatlow c_yincome2012low treat_z2012low ) ///

(life_sat= c.treatlow##c.cinema_con c_yincome2012low treat_z2012low ) if (c_yincome2012low >= -4000 & c_yincome2012low <= 4000) & syear>2011 , ind( $cmp_oprobit $cmp_oprobit) qui robust

* High-Skilled and bandwidth 2,000 Euros

xi:cmp (cinema_con= treat c_yincome2012 treat_z2012 ) ///

(life_sat= c.treat##c.cinema_con c_yincome2012 treat_z2012 ) if (c_yincome2012 >= -2000 & c_yincome2012 <= 2000) & syear>2011 , ind( $cmp_oprobit $cmp_oprobit) qui robust

* High-Skilled and bandwidth 3,000 Euros

xi:cmp (cinema_con= treat c_yincome2012 treat_z2012 ) ///

(life_sat= c.treat##c.cinema_con c_yincome2012 treat_z2012 ) if (c_yincome2012 >= -3000 & c_yincome2012 <= 3000) & syear>2011 , ind( $cmp_oprobit $cmp_oprobit) qui robust

* High-Skilled and bandwidth 4,000 Euros

xi:cmp (cinema_con= treat c_yincome2012 treat_z2012 ) ///

(life_sat= c.treat##c.cinema_con c_yincome2012 treat_z2012 ) if (c_yincome2012 >= -4000 & c_yincome2012 <= 4000) & syear>2011 , ind( $cmp_oprobit $cmp_oprobit) qui robust

*** The same for Theatre, opera and classical music performance in table 4

**Shortage occupations and bandwidth 2,000 Euros

xi:cmp (theatre= treatlow c_yincome2012low treat_z2012low ) ///

(life_sat= c.treatlow##c.theatre c_yincome2012low treat_z2012low ) if (c_yincome2012low >= -2000 & c_yincome2012low <= 2000) & syear>2011 , ind( $cmp_oprobit $cmp_oprobit) qui robust

**Shortage occupations and bandwidth 3,000 Euros

xi:cmp (theatre= treatlow c_yincome2012low treat_z2012low ) ///

(life_sat= c.treatlow##c.theatre c_yincome2012low treat_z2012low ) if (c_yincome2012low >= -3000 & c_yincome2012low <= 3000) & syear>2011 , ind( $cmp_oprobit $cmp_oprobit) qui robust

**Shortage occupations and bandwidth 4,000 Euros

xi:cmp (theatre= treatlow c_yincome2012low treat_z2012low ) ///

(life_sat= c.treatlow##c.theatre c_yincome2012low treat_z2012low ) if (c_yincome2012low >= -4000 & c_yincome2012low <= 4000) & syear>2011 , ind( $cmp_oprobit $cmp_oprobit) qui robust

* High-Skilled and bandwidth 2,000 Euros

xi:cmp (theatre= treat c_yincome2012 treat_z2012 ) ///

(life_sat= c.treat##c.theatre c_yincome2012 treat_z2012 ) if (c_yincome2012 >= -2000 & c_yincome2012 <= 2000) & syear>2011 , ind( $cmp_oprobit $cmp_oprobit) qui robust

* High-Skilled and bandwidth 3,000 Euros

xi:cmp (theatre= treat c_yincome2012 treat_z2012 ) ///

(life_sat= c.treat##c.theatre c_yincome2012 treat_z2012 ) if (c_yincome2012 >= -3000 & c_yincome2012 <= 3000) & syear>2011 , ind( $cmp_oprobit $cmp_oprobit) qui robust

* High-Skilled and bandwidth 4,000 Euros

xi:cmp (theatre= treat c_yincome2012 treat_z2012 ) ///

(life_sat= c.treat##c.theatre c_yincome2012 treat_z2012 ) if (c_yincome2012 >= -4000 & c_yincome2012 <= 4000) & syear>2011 , ind( $cmp_oprobit $cmp_oprobit) qui robust

*** Then for practicing artistic activities in table 6

**Shortage occupations and bandwidth 2,000 Euros

xi:cmp (make_arts= treatlow c_yincome2012low treat_z2012low ) ///

(life_sat= c.treatlow##c.make_arts c_yincome2012low treat_z2012low ) if (c_yincome2012low >= -2000 & c_yincome2012low <= 2000) & syear>2011 , ind( $cmp_oprobit $cmp_oprobit) qui robust

**Shortage occupations and bandwidth 3,000 Euros

xi:cmp (make_arts= treatlow c_yincome2012low treat_z2012low ) ///

(life_sat= c.treatlow##c.make_arts c_yincome2012low treat_z2012low ) if (c_yincome2012low >= -3000 & c_yincome2012low <= 3000) & syear>2011 , ind( $cmp_oprobit $cmp_oprobit) qui robust

**Shortage occupations and bandwidth 4,000 Euros

xi:cmp (make_arts= treatlow c_yincome2012low treat_z2012low ) ///

(life_sat= c.treatlow##c.make_arts c_yincome2012low treat_z2012low ) if (c_yincome2012low >= -4000 & c_yincome2012low <= 4000) & syear>2011 , ind( $cmp_oprobit $cmp_oprobit) qui robust

* High-Skilled and bandwidth 2,000 Euros

xi:cmp (make_arts= treat c_yincome2012 treat_z2012 ) ///

(life_sat= c.treat##c.make_arts c_yincome2012 treat_z2012 ) if (c_yincome2012 >= -2000 & c_yincome2012 <= 2000) & syear>2011 , ind( $cmp_oprobit $cmp_oprobit) qui robust

* High-Skilled and bandwidth 3,000 Euros

xi:cmp (make_arts= treat c_yincome2012 treat_z2012 ) ///

(life_sat= c.treat##c.make_arts c_yincome2012 treat_z2012 ) if (c_yincome2012 >= -3000 & c_yincome2012 <= 3000) & syear>2011 , ind( $cmp_oprobit $cmp_oprobit) qui robust

* High-Skilled and bandwidth 4,000 Euros

xi:cmp (make_arts= treat c_yincome2012 treat_z2012 ) ///

(life_sat= c.treat##c.make_arts c_yincome2012 treat_z2012 ) if (c_yincome2012 >= -4000 & c_yincome2012 <= 4000) & syear>2011 , ind( $cmp_oprobit $cmp_oprobit) qui robust

*** Triangle RDD with bandwidth 4,000 in table 7

gen weightslow = .

replace weightslow = (1 - abs(c_yincome2012low / 4000)) if c_yincome2012low >= -4000 & c_yincome2012low < 0

replace weightslow = (1 - abs(c_yincome2012low / 4000)) if c_yincome2012low >= 0 & c_yincome2012low <= 4000

gen weightsh = .

replace weightsh = (1 - abs(c_yincome2012 / 4000)) if c_yincome2012 >= -4000 & c_yincome2012 < 0

replace weightsh = (1 - abs(c_yincome2012 / 4000)) if c_yincome2012 >= 0 & c_yincome2012 <= 4000

**Shortage occupations and cinema

xi:cmp (cinema_con= treatlow c_yincome2012low treat_z2012low ) ///

(life_sat= c.treatlow##c.cinema_con c_yincome2012low treat_z2012low ) [aw = weightslow] if syear>2011 , ind( $cmp_oprobit $cmp_oprobit) qui robust

**Shortage occupations and theatre

xi:cmp (theatre= treatlow c_yincome2012low treat_z2012low ) ///

(life_sat= c.treatlow##c.theatre c_yincome2012low treat_z2012low ) [aw = weightslow] if syear>2011 , ind( $cmp_oprobit $cmp_oprobit) qui robust

**Shortage occupations and practicing artistic activities

xi:cmp (make_arts= treatlow c_yincome2012low treat_z2012low ) ///

(life_sat= c.treatlow##c.make_arts c_yincome2012low treat_z2012low ) [aw = weightslow] if syear>2011 , ind( $cmp_oprobit $cmp_oprobit) qui robust

**High-skilled occupations and cinema

xi:cmp (cinema_con= treat c_yincome2012 treat_z2012 ) ///

(life_sat= c.treat##c.cinema_con c_yincome2012 treat_z2012 ) [aw = weightsh] if syear>2011 , ind( $cmp_oprobit $cmp_oprobit) qui robust

**High-skilled and theatre

xi:cmp (theatre= treat c_yincome2012 treat_z2012 ) ///

(life_sat= c.treat##c.theatre c_yincome2012 treat_z2012 ) [aw = weightsh] if syear>2011 , ind( $cmp_oprobit $cmp_oprobit) qui robust

**High-skilled and practicing artistic activities

xi:cmp (make_arts= treat c_yincome2012 treat_z2012 ) ///

(life_sat= c.treat##c.make_arts c_yincome2012 treat_z2012 ) [aw = weightsh] if syear>2011 , ind( $cmp_oprobit $cmp_oprobit) qui robust

*** Fuzzy RDD Full sample Table 8 without controls

** Shortage Occupations

xi:cmp (eublue= treatlow c_yincome2012low treat_z2012low) (cinema_concerts=eublue c_yincome2012low treat_z2012low) ///

(life_sat=c.eublue##c.cinema_concerts c_yincome2012low treat_z2012low ), ind( $cmp_probit $cmp_oprobit $cmp_oprobit) robust qui

test _b[treatlow]= 0

xi:cmp (eublue= treatlow c_yincome2012low treat_z2012low) (theatre=eublue c_yincome2012low treat_z2012low) ///

(life_sat=c.eublue##c.theatre c_yincome2012low treat_z2012low ), ind( $cmp_probit $cmp_oprobit $cmp_oprobit) robust qui

test _b[treatlow]= 0

xi:cmp (eublue= treatlow c_yincome2012low treat_z2012low) (make_arts=eublue c_yincome2012low treat_z2012low) ///

(life_sat=c.eublue##c.make_arts c_yincome2012low treat_z2012low ), ind( $cmp_probit $cmp_oprobit $cmp_oprobit) robust qui

test _b[treatlow]= 0

** FRDD with controls

xi:cmp (eublue= treatlow c_yincome2012low treat_z2012low) (cinema_concerts=eublue c_yincome2012low treat_z2012low gender age age2 lnmonthly_hh_income i.pgisced97 i.mastat i.pglfs hhsize i.state) ///

(life_sat=c.eublue##c.cinema_concerts c_yincome2012low treat_z2012low gender age age2 lnmonthly_hh_income i.pgisced97 i.mastat i.pglfs hhsize i.state ), ind( $cmp_probit $cmp_oprobit $cmp_oprobit) robust qui

test _b[treatlow]= 0

xi:cmp (eublue= treatlow c_yincome2012low treat_z2012low) (theatre=eublue c_yincome2012low treat_z2012low gender age age2 lnmonthly_hh_income i.pgisced97 i.mastat i.pglfs hhsize i.state) ///

(life_sat=c.eublue##c.theatre c_yincome2012low treat_z2012low gender age age2 lnmonthly_hh_income i.pgisced97 i.mastat i.pglfs hhsize i.state ), ind( $cmp_probit $cmp_oprobit $cmp_oprobit) robust ui

test _b[treatlow]= 0

xi:cmp (eublue= treatlow c_yincome2012low treat_z2012low) (make_arts=eublue c_yincome2012low treat_z2012low gender age age2 lnmonthly_hh_income i.pgisced97 i.mastat i.pglfs hhsize i.state) ///

(life_sat=c.eublue##c.make_arts c_yincome2012low treat_z2012low gender age age2 lnmonthly_hh_income i.pgisced97 i.mastat i.pglfs hhsize i.state ), ind( $cmp_probit $cmp_oprobit $cmp_oprobit) robust qui

test _b[treatlow]= 0

*** High Skilled

xi:cmp (eublue= treat c_yincome2012 treat_z2012) (cinema_concerts=eublue c_yincome2012 treat_z2012) ///

(life_sat=c.eublue##c.cinema_concerts c_yincome2012 treat_z2012 ) , ind( $cmp_probit $cmp_oprobit $cmp_oprobit) robust qui

test _b[treat]= 0

xi:cmp (eublue= treat c_yincome2012 treat_z2012) (theatre=eublue c_yincome2012 treat_z2012) ///

(life_sat=c.eublue##c.theatre c_yincome2012 treat_z2012 ), ind( $cmp_probit $cmp_oprobit $cmp_oprobit) robust qui

test _b[treat]= 0

xi:cmp (eublue= treat c_yincome2012 treat_z2012) (make_arts=eublue c_yincome2012 treat_z2012) ///

(life_sat=c.eublue##c.make_arts c_yincome2012 treat_z2012 ) if syear>=2017 , ind( $cmp_probit $cmp_oprobit $cmp_oprobit) robust qui

test _b[treat]= 0

*** High skilled with controls

xi:cmp (eublue= treat c_yincome2012 treat_z2012) (cinema_concerts=eublue c_yincome2012 treat_z2012 gender age age2 lnmonthly_hh_income i.pgisced97 i.mastat i.pglfs hhsize i.state) ///

(life_sat=c.eublue##c.cinema_concerts c_yincome2012 treat_z2012 gender age age2 lnmonthly_hh_income i.pgisced97 i.mastat i.pglfs hhsize i.state) , ind( $cmp_probit $cmp_oprobit $cmp_oprobit) robust qui

test _b[treat]= 0

xi:cmp (eublue= treat c_yincome2012 treat_z201) (theatre=eublue c_yincome2012 treat_z2012 gender age age2 lnmonthly_hh_income i.pgisced97 i.mastat i.pglfs hhsize i.state) ///

(life_sat=c.eublue##c.theatre c_yincome2012 treat_z2012 gender age age2 lnmonthly_hh_income i.pgisced97 i.mastat i.pglfs hhsize i.state), ind( $cmp_probit $cmp_oprobit $cmp_oprobit) robust qui

test _b[treat]= 0

xi:cmp (eublue= treat c_yincome2012 treat_z201) (make_arts=eublue c_yincome2012 treat_z2012 gender age age2 lnmonthly_hh_income i.pgisced97 i.mastat i.pglfs hhsize i.state) ///

(life_sat=c.eublue##c.make_arts c_yincome2012 treat_z2012 gender age age2 lnmonthly_hh_income i.pgisced97 i.mastat i.pglfs hhsize i.state), ind( $cmp_probit $cmp_oprobit $cmp_oprobit) robust qui

test _b[treat]= 0

** The same with table 9 but with bandwidth of 4,000

** Shortage Occupations

xi:cmp (eublue= treatlow c_yincome2012low treat_z2012low) (cinema_concerts=eublue c_yincome2012low treat_z2012low) ///

(life_sat=c.eublue##c.cinema_concerts c_yincome2012low treat_z2012low ) if (c_yincome2012low >= -4000 & c_yincome2012low <= 4000) , ind( $cmp_probit $cmp_oprobit $cmp_oprobit) robust qui

test _b[treatlow]= 0

xi:cmp (eublue= treatlow c_yincome2012low treat_z2012low) (theatre=eublue c_yincome2012low treat_z2012low) ///

(life_sat=c.eublue##c.theatre c_yincome2012low treat_z2012low ) if (c_yincome2012low >= -4000 & c_yincome2012low <= 4000) , ind( $cmp_probit $cmp_oprobit $cmp_oprobit) robust qui

test _b[treatlow]= 0

xi:cmp (eublue= treatlow c_yincome2012low treat_z2012low) (make_arts=eublue c_yincome2012low treat_z2012low) ///

(life_sat=c.eublue##c.make_arts c_yincome2012low treat_z2012low ) if (c_yincome2012low >= -4000 & c_yincome2012low <= 4000) , ind( $cmp_probit $cmp_oprobit $cmp_oprobit) robust qui

test _b[treatlow]= 0

*** High skilled

xi:cmp (eublue= treat c_yincome2012 treat_z2012) (cinema_concerts=eublue c_yincome2012 treat_z2012) ///

(life_sat=c.eublue##c.cinema_concerts c_yincome2012 treat_z2012 ) if (c_yincome2012 >= -4000 & c_yincome2012 <= 4000) & syear>=2017, ind( $cmp_probit $cmp_oprobit $cmp_oprobit) robust qui

test _b[treat]= 0

xi:cmp (eublue= treat c_yincome2012 treat_z2012) (theatre=eublue c_yincome2012 treat_z2012) ///

(life_sat=c.eublue##c.theatre c_yincome2012 treat_z2012 ) if (c_yincome2012 >= -4000 & c_yincome2012 <= 4000) & syear>=2017, ind( $cmp_probit $cmp_oprobit $cmp_oprobit) robust qui

test _b[treat]= 0

xi:cmp (eublue= treat c_yincome2012 treat_z2012) (make_arts=eublue c_yincome2012 treat_z2012) ///

(life_sat=c.eublue##c.make_arts c_yincome2012 treat_z2012 ) if (c_yincome2012 >= -4000 & c_yincome2012 <= 4000) & syear>=2017 , ind( $cmp_probit $cmp_oprobit $cmp_oprobit) robust qui

test _b[treat]= 0

*** Table 10 Ordered Probit SURE with controls

xi:gsem (cinema_concerts<- eublue gender age age2 lnmonthly_hh_income i.pgisced97 i.mastat i.pglfs hhsize i.state , oprobit ) ///

(life_sat<- c.eublue##c.cinema_concerts gender age age2 lnmonthly_hh_income i.pgisced97 i.mastat i.pglfs hhsize i.state , oprobit ) , vce(robust)

xi:gsem (theatre<- eublue gender age age2 lnmonthly_hh_income i.pgisced97 i.mastat i.pglfs hhsize i.state , oprobit ) ///

(life_sat<- c.eublue##c.theatre gender age age2 lnmonthly_hh_income i.pgisced97 i.mastat i.pglfs hhsize i.state , oprobit ) , vce(robust)

xi:gsem (make_arts<- eublue gender age age2 lnmonthly_hh_income i.pgisced97 i.mastat i.pglfs hhsize i.state , oprobit ) ///

(life_sat<- c.eublue##c.make_arts gender age age2 lnmonthly_hh_income i.pgisced97 i.mastat i.pglfs hhsize i.state , oprobit ) , vce(robust)

*** Similar table 11 sharp RDD with bandwidth 4,000

**Cinema

xi:cmp (cinema_con= treatlow c_yincome2012low treat_z2012low ) ///

(life_sat= c.treatlow##c.cinema_con c_yincome2012low treat_z2012low ) if (c_yincome2012low >= -4000 & c_yincome2012low <= 4000) & syear==2011 , ind( $cmp_oprobit $cmp_oprobit) qui robust

*** theatre

xi:cmp (theatre= treatlow c_yincome2012low treat_z2012low ) ///

(life_sat= c.treatlow##c.theatre c_yincome2012low treat_z2012low ) if (c_yincome2012low >= -4000 & c_yincome2012low <= 4000) & syear==2011 , ind( $cmp_oprobit $cmp_oprobit) qui robust

** practicing artistic activities

xi:cmp (make_arts= treatlow c_yincome2012low treat_z2012low ) ///

(life_sat= c.treatlow##c.make_arts c_yincome2012low treat_z2012low ) if (c_yincome2012low >= -4000 & c_yincome2012low <= 4000) & syear==2011 , ind( $cmp_oprobit $cmp_oprobit) qui robust

**Cinema

xi:cmp (cinema_con= treat c_yincome2012 treat_z2012 ) ///

(life_sat= c.treat##c.cinema_con c_yincome2012 treat_z2012 ) if (c_yincome2012 >= -4000 & c_yincome2012 <= 4000) & syear==2011 , ind( $cmp_oprobit $cmp_oprobit) qui robust

*** theatre

xi:cmp (theatre= treat c_yincome2012 treat_z2012 ) ///

(life_sat= c.treat##c.theatre c_yincome2012 treat_z2012 ) if (c_yincome2012 >= -4000 & c_yincome2012 <= 4000) & syear==2011 , ind( $cmp_oprobit $cmp_oprobit) qui robust

** practicing artistic activities

xi:cmp (make_arts= treat c_yincome2012 treat_z2012 ) ///

(life_sat= c.treat##c.make_arts c_yincome2012 treat_z2012 ) if (c_yincome2012 >= -4000 & c_yincome2012 <= 4000) & syear==2011 , ind( $cmp_oprobit $cmp_oprobit) qui robust

**** Table 12 Gender

*** MALES

** Shortage Occupations

xi:cmp (eublue= treatlow c_yincome2012low treat_z2012low) (cinema_concerts=eublue c_yincome2012low treat_z2012low) ///

(life_sat=c.eublue##c.cinema_concerts c_yincome2012low treat_z2012low ) if gender==1, ind( $cmp_probit $cmp_oprobit $cmp_oprobit) robust qui

test _b[treatlow]= 0

xi:cmp (eublue= treatlow c_yincome2012low treat_z2012low) (theatre=eublue c_yincome2012low treat_z2012low) ///

(life_sat=c.eublue##c.theatre c_yincome2012low treat_z2012low ) if gender==1, ind( $cmp_probit $cmp_oprobit $cmp_oprobit) robust qui

test _b[treatlow]= 0

xi:cmp (eublue= treatlow c_yincome2012low treat_z2012low) (make_arts=eublue c_yincome2012low treat_z2012low) ///

(life_sat=c.eublue##c.make_arts c_yincome2012low treat_z2012low ) if gender==1, ind( $cmp_probit $cmp_oprobit $cmp_oprobit) robust qui

test _b[treatlow]= 0

*** High Skilled

xi:cmp (eublue= treat c_yincome2012 treat_z2012) (cinema_concerts=eublue c_yincome2012 treat_z2012) ///

(life_sat=c.eublue##c.cinema_concerts c_yincome2012 treat_z2012 ) if gender==1 , ind( $cmp_probit $cmp_oprobit $cmp_oprobit) robust qui

test _b[treat]= 0

xi:cmp (eublue= treat c_yincome2012 treat_z2012) (theatre=eublue c_yincome2012 treat_z2012) ///

(life_sat=c.eublue##c.theatre c_yincome2012 treat_z2012 ) if gender==1, ind( $cmp_probit $cmp_oprobit $cmp_oprobit) robust qui

test _b[treat]= 0

xi:cmp (eublue= treat c_yincome2012 treat_z2012) (make_arts=eublue c_yincome2012 treat_z2012) ///

(life_sat=c.eublue##c.make_arts c_yincome2012 treat_z2012 ) if gender==1 , ind( $cmp_probit $cmp_oprobit $cmp_oprobit) robust qui

test _b[treat]= 0

** FEMALES

** Shortage Occupations

xi:cmp (eublue= treatlow c_yincome2012low treat_z2012low) (cinema_concerts=eublue c_yincome2012low treat_z2012low) ///

(life_sat=c.eublue##c.cinema_concerts c_yincome2012low treat_z2012low ) if gender==2, ind( $cmp_probit $cmp_oprobit $cmp_oprobit) robust qui

test _b[treatlow]= 0

xi:cmp (eublue= treatlow c_yincome2012low treat_z2012low) (theatre=eublue c_yincome2012low treat_z2012low) ///

(life_sat=c.eublue##c.theatre c_yincome2012low treat_z2012low ) if gender==2, ind( $cmp_probit $cmp_oprobit $cmp_oprobit) robust qui

test _b[treatlow]= 0

xi:cmp (eublue= treatlow c_yincome2012low treat_z2012low) (make_arts=eublue c_yincome2012low treat_z2012low) ///

(life_sat=c.eublue##c.make_arts c_yincome2012low treat_z2012low ) if gender==2, ind( $cmp_probit $cmp_oprobit $cmp_oprobit) robust qui

test _b[treatlow]= 0

*** High Skilled

xi:cmp (eublue= treat c_yincome2012 treat_z2012) (cinema_concerts=eublue c_yincome2012 treat_z2012) ///

(life_sat=c.eublue##c.cinema_concerts c_yincome2012 treat_z2012 ) if gender==2 , ind( $cmp_probit $cmp_oprobit $cmp_oprobit) robust qui

test _b[treat]= 0

xi:cmp (eublue= treat c_yincome2012 treat_z2012) (theatre=eublue c_yincome2012 treat_z2012) ///

(life_sat=c.eublue##c.theatre c_yincome2012 treat_z2012 ) if gender==2, ind( $cmp_probit $cmp_oprobit $cmp_oprobit) robust qui

test _b[treat]= 0

xi:cmp (eublue= treat c_yincome2012 treat_z2012) (make_arts=eublue c_yincome2012 treat_z2012) ///

(life_sat=c.eublue##c.make_arts c_yincome2012 treat_z2012 ) if gender==2 , ind( $cmp_probit $cmp_oprobit $cmp_oprobit) robust qui

test _b[treat]= 0

** age

** age <45 shortage occupations

xi:cmp (eublue= treatlow c_yincome2012low treat_z2012low) (cinema_concerts=eublue c_yincome2012low treat_z2012low) ///

(life_sat=c.eublue##c.cinema_concerts c_yincome2012low treat_z2012low ) if age<45, ind( $cmp_probit $cmp_oprobit $cmp_oprobit) robust qui

test _b[treatlow]= 0

xi:cmp (eublue= treatlow c_yincome2012low treat_z2012low) (theatre=eublue c_yincome2012low treat_z2012low) ///

(life_sat=c.eublue##c.theatre c_yincome2012low treat_z2012low ) if age<45, ind( $cmp_probit $cmp_oprobit $cmp_oprobit) robust qui

test _b[treatlow]= 0

xi:cmp (eublue= treatlow c_yincome2012low treat_z2012low) (make_arts=eublue c_yincome2012low treat_z2012low) ///

(life_sat=c.eublue##c.make_arts c_yincome2012low treat_z2012low ) if age<45, ind( $cmp_probit $cmp_oprobit $cmp_oprobit) robust qui

test _b[treatlow]= 0

** age+44 shortage occupations

xi:cmp (eublue= treatlow c_yincome2012low treat_z2012low) (cinema_concerts=eublue c_yincome2012low treat_z2012low) ///

(life_sat=c.eublue##c.cinema_concerts c_yincome2012low treat_z2012low ) if age>=45, ind( $cmp_probit $cmp_oprobit $cmp_oprobit) robust qui

test _b[treatlow]= 0

xi:cmp (eublue= treatlow c_yincome2012low treat_z2012low) (theatre=eublue c_yincome2012low treat_z2012low) ///

(life_sat=c.eublue##c.theatre c_yincome2012low treat_z2012low ) if age>=45, ind( $cmp_probit $cmp_oprobit $cmp_oprobit) robust qui

test _b[treatlow]= 0

xi:cmp (eublue= treatlow c_yincome2012low treat_z2012low) (make_arts=eublue c_yincome2012low treat_z2012low) ///

(life_sat=c.eublue##c.make_arts c_yincome2012low treat_z2012low ) if age>=45, ind( $cmp_probit $cmp_oprobit $cmp_oprobit) robust qui

test _b[treatlow]= 0

** age <45 high-skilled

xi:cmp (eublue= treat c_yincome2012 treat_z2012) (cinema_concerts=eublue c_yincome2012 treat_z2012) ///

(life_sat=c.eublue##c.cinema_concerts c_yincome2012 treat_z2012 ) if age<45 , ind( $cmp_probit $cmp_oprobit $cmp_oprobit) robust qui

test _b[treat]= 0

xi:cmp (eublue= treat c_yincome2012 treat_z2012) (theatre=eublue c_yincome2012 treat_z2012) ///

(life_sat=c.eublue##c.theatre c_yincome2012 treat_z2012 ) if age<45, ind( $cmp_probit $cmp_oprobit $cmp_oprobit) robust qui

test _b[treat]= 0

xi:cmp (eublue= treat c_yincome2012 treat_z2012) (make_arts=eublue c_yincome2012 treat_z2012) ///

(life_sat=c.eublue##c.make_arts c_yincome2012 treat_z2012 ) if age<45 , ind( $cmp_probit $cmp_oprobit $cmp_oprobit) robust qui

test _b[treat]= 0

** age+44 high-skilled

xi:cmp (eublue= treat c_yincome2012 treat_z2012) (cinema_concerts=eublue c_yincome2012 treat_z2012) ///

(life_sat=c.eublue##c.cinema_concerts c_yincome2012 treat_z2012 ) if age>=45 , ind( $cmp_probit $cmp_oprobit $cmp_oprobit) robust qui

test _b[treat]= 0

xi:cmp (eublue= treat c_yincome2012 treat_z2012) (theatre=eublue c_yincome2012 treat_z2012) ///

(life_sat=c.eublue##c.theatre c_yincome2012 treat_z2012 ) if age>=45, ind( $cmp_probit $cmp_oprobit $cmp_oprobit) robust qui

test _b[treat]= 0

xi:cmp (eublue= treat c_yincome2012 treat_z2012) (make_arts=eublue c_yincome2012 treat_z2012) ///

(life_sat=c.eublue##c.make_arts c_yincome2012 treat_z2012 ) if age>=45 , ind( $cmp_probit $cmp_oprobit $cmp_oprobit) robust qui

test _b[treat]= 0
